# Ectopic expression of *BOTRYTIS SUSCEPTIBLE1* reveals its function as a positive regulator of wound-induced cell death and plant susceptibility to *Botrytis*

**DOI:** 10.1093/plcell/koac206

**Published:** 2022-08-10

**Authors:** Fuqiang Cui, Xiaoxiao Li, Wenwu Wu, Wenbo Luo, Ying Wu, Mikael Brosché, Kirk Overmyer

**Affiliations:** State Key Laboratory of Subtropical Silviculture, Zhejiang A&F University, Hangzhou 311300, China; State Key Laboratory of Subtropical Silviculture, Zhejiang A&F University, Hangzhou 311300, China; State Key Laboratory of Subtropical Silviculture, Zhejiang A&F University, Hangzhou 311300, China; State Key Laboratory of Subtropical Silviculture, Zhejiang A&F University, Hangzhou 311300, China; State Key Laboratory of Subtropical Silviculture, Zhejiang A&F University, Hangzhou 311300, China; Faculty of Biological and Environmental Sciences, Organismal and Evolutionary Biology Research Program, Viikki Plant Science Centre, University of Helsinki, Helsinki FI-00014, Finland; Faculty of Biological and Environmental Sciences, Organismal and Evolutionary Biology Research Program, Viikki Plant Science Centre, University of Helsinki, Helsinki FI-00014, Finland

## Abstract

Programmed cell death (PCD) is integral to plant life and required for stress responses, immunity, and development. Our understanding of the regulation of PCD is incomplete, especially concerning regulators involved in multiple divergent processes. The *botrytis-susceptible* (*bos1*) mutant of Arabidopsis is highly susceptible to fungal infection by *Botrytis cinerea* (*Botrytis*). BOS1 (also known as MYB108) regulates cell death propagation during plant responses to wounding. The *bos1-1* allele contains a T-DNA insertion in the 5′-untranslated region upstream of the start codon. This insertion results in elevated expression of *BOS1/MYB108*. We used clustered regularly interspaced short palindromic repeats (CRISPR) and CRISPR-associated nuclease 9 (Cas9) system (CRISPR/Cas9) to create new *bos1* alleles with disrupted exons, and found that these lines lacked the typical *bos1-1* wounding and *Botrytis* phenotypes. They did exhibit reduced fertility, as was previously observed in other *bos1* alleles. Resequencing of the *bos1-1* genome confirmed the presence of a *mannopine synthase* (*MAS*) promoter at the T-DNA left border. Expression of the *BOS1* gene under control of the *MAS* promoter in wild-type plants conferred the characteristic phenotypes of *bos1-1*: *Botrytis* sensitivity and response to wounding. Multiple overexpression lines demonstrated that *BOS1* was involved in regulation of cell death propagation in a dosage-dependent manner. Our data indicate that *bos1-1* is a gain-of-function mutant and that BOS1 function in regulation of fertility and *Botrytis* response can both be understood as misregulated cell death.

## Teaser

*MYB108/BOS1* enhances cell death in the gain-of-function mutant *bos1-1* after fungal infection and mechanical wounding.

IN A NUTSHELL **Background:** Programmed cell death (PCD) is essential for proper plant development and life. Following inititation of PCD in plant cells, cell death can spread to surrounding cells in a process called propagation. PCD is finely controlled and benefits plants in ways such as killing cells infected by pathogens, closing wounds, or sculpting forms during development. *BOS1* (also known as *MYB108*) encodes a transcription factor with an important role in plant PCD. Improper function of *BOS1* results in the uncontrolled spread of cell death, indicating that *BOS1* regulates PCD propagation. Previous studies showed that loss of *BOS1* action results in uncontrolled cell death, suggesting that *BOS1* acts as a suppressor of cell death.

**Questions:** Here we revisit the genetics of *BOS1* mutants using new experimental tools not available in previous studies and ask: How does *BOS1* regulate the propagation of PCD, and how does the particular site of T-DNA insertion affect *BOS1* function?

**Findings:** We show that *BOS1* promotes, rather than inhibits, the propagation of cell death. The previous studies used a single Arabidopsis mutant (*bos1-1*), which contains a T-DNA insertion in the 5′-untranslated region. This T-DNA insertion did not disrupt *BOS1* as expected, but instead, it altered the activation of *BOS1* upon wounding and pathogen attack due to sequences in the T-DNA that regulate gene activation. T-DNAs containing similar regulatory sequences were used to construct insertion mutant collections that are widely used in plant research. Other mutants with such T-DNAs may also result in changed activation of nearby genes, as was seen in *bos1-1*. Thus, the regulatory sequence of a T-DNA insertion must be taken into consideration when evaluating results of studies using this type of insertion mutants.

**Next steps:** To better define the role of BOS1 in PCD, we aim to identify the target genes that are regulated by *BOS1*. We also plan to search for other MYB transcription factors that potentially act together with BOS1 to regulate PCD.

## Introduction

Programmed cell death (PCD) is a finely tuned process that occurs during plant–pathogen interactions and plant development. Generally, PCD has three stages: initiation, propagation, and containment (McCabe, 2013; Van Hautegem et al., 2015). In *Arabidopsis thaliana* (Arabidopsis), regulators of the initiation stage have been identified primarily through analysis of lesion-mimic mutants that spontaneously develop leaf necrosis (Lorrain et al., 2003; Bruggeman et al., 2015). Regulators of cell death propagation and containment have been identified through mapping of lesion-mimic mutants (Bruggeman et al., 2015). In such mutants, initiation of PCD is followed by uncontained spread of cell death that can consume the entire leaf (Lorrain et al., 2003). These mutants have been instrumental in elucidating the mechanism of cell death, including regulation by plant hormones such as salicylic acid and jasmonates (Bruggeman et al., 2015). However, our understanding of the signals leading to the propagation and subsequent containment of cell death remains incomplete. One open question in plant PCD research is to what extent do pathogen activated and developmental PCD have overlapping regulatory mechanisms and execution (Huysmans et al., 2017).

Mechanical injury results in cell death in tissue adjacent to the wound in order to re-establish the integument (Bostock and Stermer, 1989; McCabe, 2013; Cui et al., 2013; Lakimova and Woltering, 2018). Uncontained, abscisic acid dependent, PCD propagation was found in *botrytis-susceptible1-1* (*bos1-1*; Cui et al., 2013), a mutant allele of *BOS1*/*MYB108* (*AT3G06490*; Mengiste et al., 2003). PCD propagation is enhanced in *bos1-1* following PCD initiation by pathogen infection or mechanical injury (Cui et al., 2013, 2019). This makes *bos1-1* a useful model to study the mechanism of PCD propagation (McCabe, 2013). BOS1 is an R2R3 MYB transcription factor that was functionally characterized in the seminal paper by Mengiste et al. (2003) through the analysis of the *bos1-1* mutant, which is extremely susceptible to the necrotrophic fungal pathogen *Botrytis cinerea*. Results from subsequent studies utilizing the *bos1-1* mutant demonstrated that *BOS1* is a key regulator of cell death in plant–pathogen interactions, especially those involving necrotrophic fungi (Kraepiel et al., 2011; Cui et al., 2013, 2019). BOS1 levels are regulated by BOTRYTIS SUSCEPTIBLE1 INTERACTOR (BOI), an E3 ligase that attenuates stress-induced cell death in plants (Luo et al., 2010).The *bos1-1* allele was isolated from a T-DNA mutant pool that was later released to the community as the SAIL collection (McElver et al., 2001; Sessions et al., 2002). The *bos1-1* line was genetically characterized as a recessive loss-of-function mutant, although the T-DNA insertion is located in the 5′-untranslated region (5′-UTR) just upstream from the start codon and results in an increased *BOS1* transcript level (Mengiste et al., 2003).

Aside from regulating stress responses, PCD is also indispensable for plant development, for example, formation and release of pollen (Mandaokar and Browse, 2009; Daneva et al., 2016; Xu et al., 2019). To study the role of *BOS1*/*MYB108* in anther development, three mutant alleles with T-DNA insertions in the first intron were used (Mandaokar and Browse, 2009). These mutants display reduced male fertility, lower pollen viability, and delayed anther dehiscence; however, their stress responses remain untested.

Due to differences in the BOS1 transcript level observed in various *bos1* alleles used in the study of pathogen induced and developmental PCD, it is difficult to assess the precise role of this transcription factor in both types of cell death. Furthermore, the T-DNA insertion sites within the existing alleles of *bos1* are located either in introns (Mandaokar and Browse, 2009) or in the 5′-UTR (*bos1-1*; Mengiste et al., 2003), further complicating interpretation of BOS1 function in the regulation of cell death.

Here we have generated new mutant alleles of *BOS1* with disrupted exons and present evidence that *BOS1* is a positive regulator of cell death.

## Results

### *Botrytis* and wounding responses in new *bos1* alleles made with CRISPR/Cas9

**Figure 1:**
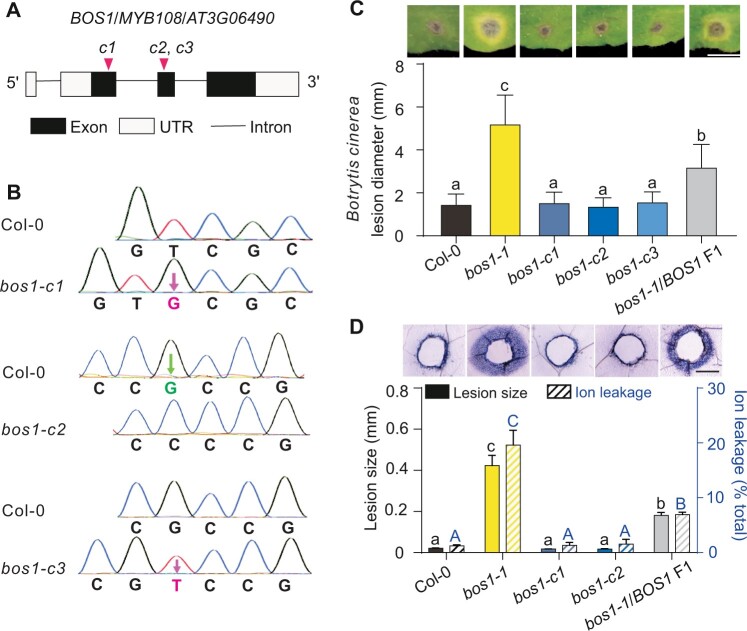
New *bos1* alleles created with CRISPR/Cas9 did not exhibit typical *bos1-1* phenotypes. A, Schematic diagram of the new *bos1* insertion and deletion alleles. The gRNA positions are indicated with magenta triangles, and the new *bos1* alleles made with the CRISPR/Cas9 system are designated *bos1-c1*, *bos1-c2*, and *bos1-c3* (abbreviated here as *c1*, *c2*, and *c3*). B, CRISPR/Cas9-induced changes in the indicated mutants. Single base insertions (magenta characters) and deletions (green characters) were detected with Sanger sequencing. These frame shifts resulted in predicted short missense sequences and truncated proteins with lengths approximately one-fourth the length of the wild-type BOS1 polypeptide. See Supplemental Figure S1. C, Disease symptoms and size of lesions caused by infection with *B. cinerea*. Droplets of a conidial suspension (3 μL, 2 × 10^5^ spores mL^−1^) were inoculated onto fully expanded leaves of the indicated genotypes. Symptoms were photographed at 3 dpi. *Botrytis*-induced lesion size was measured from photographs using ImageJ. Bars represent means ± se (three independent biological replicates, each consisting of leaves from five individual plants; *n* ≥ 40 leaves total). Letters above the bars indicate significance groups (*P* < 0.05; one-way ANOVA, Supplemental File S1). Bar = 0.5 cm. D, Wound-induced cell-death spread. Leaves punctured with a toothpick were stained with trypan blue to visualize dead tissue at 4 dpw. Representative photos are shown to illustrate the dead tissue around the wounds. The extent of cell death spread was quantified by measuring the distance from the wound edge to the outer border of the area of cell death (black bars). Bars represent mean ± se (three biological repeats, each analyzing wounds from five individual leaves; *n* = 36 total). Ion leakage (blue striped bars) from wounded leaves was measured at 5 dpw and is expressed as percentage of total ions, determined after disrupting all cell membranes by freezing. Bars represent means ± se (three biological repeats; three leaves were combined as one sample; *n* = 12 samples in total). Letters above the bars indicate significance groups (*P* < 0.05; one-way ANOVA, Supplemental File S1); lower case letters, wound-induced lesion size; upper case letters, ion leakage. Bar = 0.5 mm.

Genome editing allows the generation of precisely targeted mutations (Jiang et al., 2013; Xing et al., 2014). We used the CRISPR/Cas9 system to create three *BOS1* loss-of-function alleles, targeting the first and second exons (*bos1-c1* to *-c3*; Figure 1A). These mutations caused frame shifts resulting in the predicted truncated proteins (Figure 1B; Supplemental Figure S1). Strikingly, none of these mutants phenocopied *bos1-1* when challenged by *Botrytis* infection or wounding. *Botrytis-*induced lesion size and wound-induced cell death spread in these mutants were similar to those observed in the wild-type plants, whereas the *bos1-1* mutant characteristically exhibited large lesions (Figure 1, C and D). The discrepancy between the phenotypes of *bos1-1* and the new mutants suggested that *bos1-1* is not a loss-of-function mutant.

**Figure 2:**
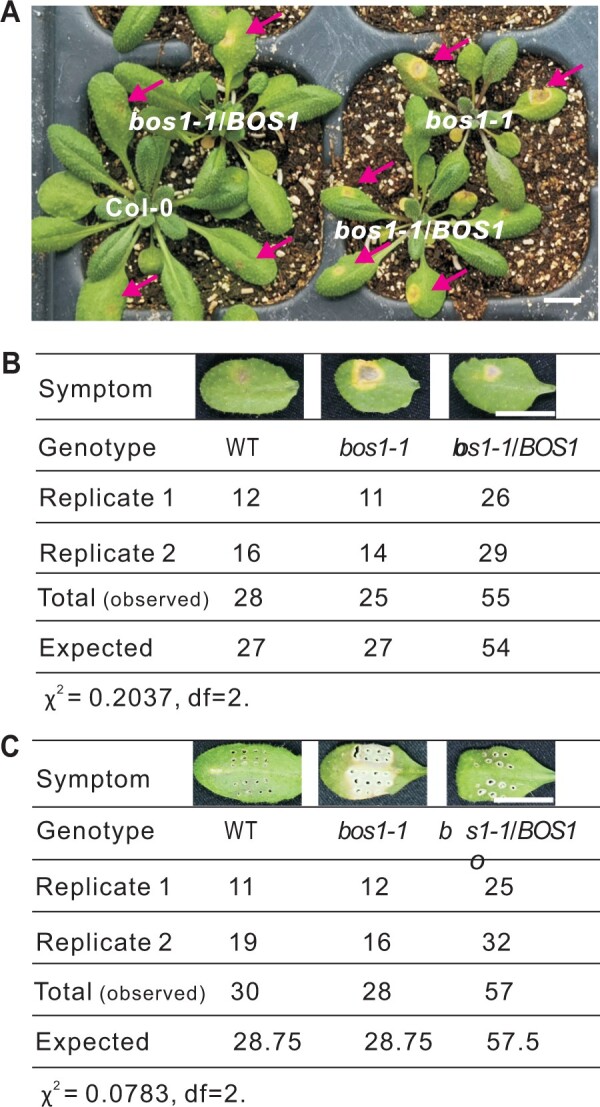
Segregation of phenotypes in F_2_ plants in response to *Botrytis* infection and wounding. A, Plant symptoms caused by *Botrytis* infection. Arrows point to developing lesions. Bar = 1 cm. B and C, The number of F_2_ individuals exhibiting the indicated symptoms upon *Botrytis* infection (B) or wounding (C). The genotypes of several plants were confirmed by PCR and are presented as representative symptoms. F_2_ individuals with similar symptoms were counted and the number of individuals in each category is listed. A model with co-dominant inheritance in *bos1-1* was used as the null hypothesis for the *χ*^2^-test. For both wound and *Botrytis* responses *χ*^2^ < 5.99, null hypothesis cannot be rejected. Bar = 1 cm.

To explore this hypothesis, *bos1-1/BOS1* plants were generated and tested upon wounding and infection with *Botrytis*. Heterozygous *bos1-1/BOS1* plants exhibited phenotypes that were intermediate between those observed in wild-type and the *bos1-1* mutant. Both the extent of wounding-induced runaway cell death and the size of *Botrytis*-induced lesions observed in *bos1-1/BOS1* plants were significantly larger than those in wild-type plants but smaller than those of *bos1-1* (Figure 1, C and D). In addition, the distribution of *Botrytis*- and wounding-induced lesions was tested in an F_2_ population derived from multiple *bos1-1*/*BOS1* F_1_ individuals. For both treatments, ∼25% of tested F_2_ individuals exhibited *bos1*-*1*-like symptoms; 50% had phenotypes of *bos1-1/BOS1* plants; and 25% had wild-type characteristics, fitting a 1:2:1 segregation ratio. Genotyping of F_2_ plants revealed a correlation between genotype and phenotype. Plants exhibiting large *Botrytis-*induced lesions were *bos1-1* homozygotes, and those with intermediate-sized lesions were heterozygotes (Figure 2). Taken together, based on the genetics in the F_1_ and F_2_ generations, we conclude that *bos1-1* is a co-dominant gain-of-function mutant.

### CRISPR/Cas9-induced *bos1* alleles are impaired in fertility

**Figure 3:**
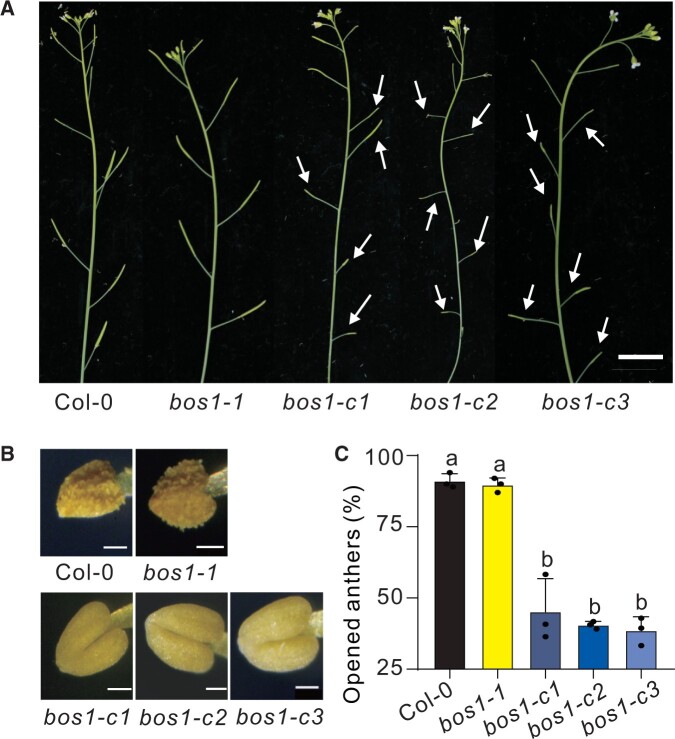
CRISPR/Cas9-induced *bos1* loss-of-function lines were impaired in pollen release. A, The *bos1* alleles created with CRISPR/Cas9 (*bos1-c1* to *bos1-c3*) resulted in impaired fertility. White arrows indicate siliques with reduced seed production. Delayed flower senescence was also apparent in the *bos1* CRISPR/Cas9-induced alleles. Bar = 1 cm. B, Anthers of the CRISPR/Cas9-induced loss-of-function alleles of *bos1* exhibited delayed dehiscence. Anthers were detached from flowers at the same developmental stage (floral stage 14 according to stages defined by Sanders et al. 1999). Bar = 50 μm. C, The proportion of open anthers in the indicated genotypes. Ten flowers of each genotype at floral stage 14 were measured. Bars represent mean ± se from three independent biological repeats. Letters above bars indicate significant differences between groups (*P* ≤ 0.05; one-way ANOVA, Supplemental File S1). Transcript levels for *BOS1* in publicly available development and stress experiments are shown in Supplemental Figure S4 and further characterization of stress responses in the new *bos1* CRISPR/Cas9-induced alleles in Supplemental Figures S5–S7.

CRISPR/Cas9-induced loss-of-function alleles exhibited strong deficiencies in fertility, having siliques with reduced size and delayed flower senescence (Figure 3A). This finding is consistent with the reduced fertility phenotypes previously observed in *bos1* mutants having T-DNAs insertions in introns (Xu et al., 2019; Mandaokar and Browse, 2009). In these previous studies, fewer anthers in *bos1* mutants had undergone dehiscence, suggesting that the impaired fertility of *bos1* is due to deficient or delayed pollen release (Xu et al., 2019). Examination of the anthers of the new CRISPR/Cas9-induced loss-of-function alleles revealed a significant reduction or delay in pollen release as compared to wild-type (Figure 3, B and C). In contrast, *bos1-1* fertility and anther dehiscence were comparable to that of wild-type plants (Figure 3). These findings further suggest *bos1-1* is not a loss-of-function mutant.

### The *bos1-1* allele is a gain-of-function allele due to an increased *BOS1* transcript level

**Figure 4:**
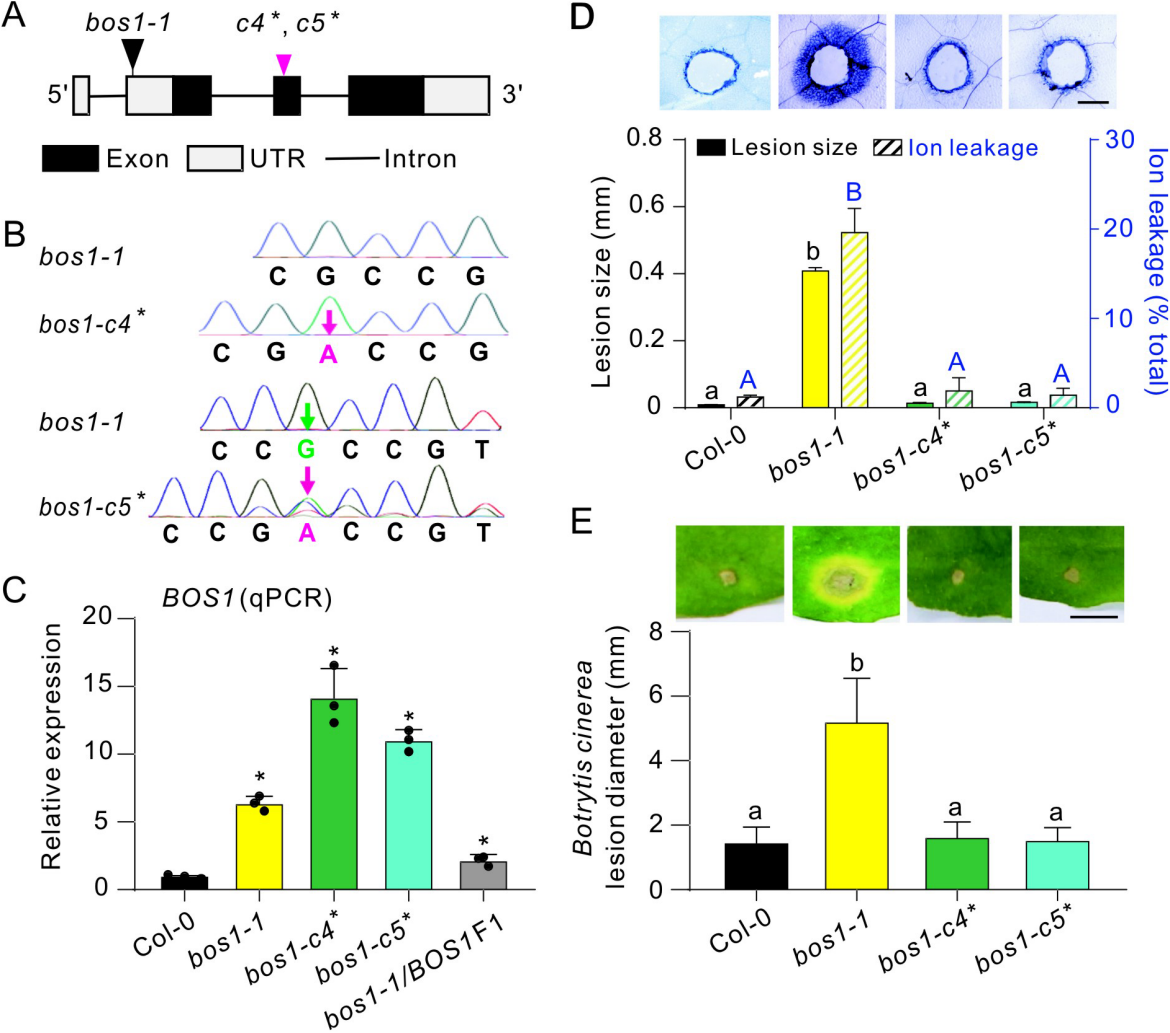
Phenotypes of *bos1-1* were suppressed by introduction of exon-disrupting alleles in *BOS1.* A, Schematic diagram of the new intragenic double mutants bos1-c4* and bos1-c5* created using the CRISPR/Cas9 system in the *bos1-1* background. These alleles had both the T-DNA insertions of *bos1-1* and the frame shift-inducing SNPs in the second exon of *BOS1*. The black triangle indicates the T-DNA insertion site in *bos1-1*. The magenta triangle indicates the start of the frame shifts in bos1-c4* and *bos1*-c5* (c4*, c5*). See Supplemental Figure S1 for the predicted amino acid sequence of the truncated proteins encoded by bos1-c4* and *bos1*-c5*. B, Single base insertions (magenta characters) and deletions (green characters) were detected with Sanger sequencing. The adenine insertion in bos1-c4* was homozygous, while in bos1-c5* there were two changes, the insertion of an adenine and the deletion of a guanine. C, Relative expression of *BOS1* in the indicated genotypes. Fully expanded leaves of 24-day-old plants were used for qPCR. Data are shown as mean ± sd (*n* = 3 technical replicates, two biological replicates showed similar results). Asterisks above the bars indicate means that are significantly different from wild-type Col-0 (*P* < 0.05; *t* test, two-sided, Supplemental File S1). D, Wounding-induced cell-death spread visualized with trypan blue staining. Following puncture of leaves with a toothpick, the distance from the wound edge to the outer border of the area of cell death was measured (black bars). Representative photos illustrate the dead tissues around the toothpick-puncture wounds. Bars represent mean ± se (three biological repeats; each analyzing wounds from five individual leaves; *n* = 12 in total). Ion leakage (blue striped bars) from wounded leaves was measured at 5 dpw and is expressed as percentage of total ions, determined after disrupting all membranes by freezing. Bars represent mean ± se (three biological repeats; three leaves were combined as one sample; *n* = 12 samples in total). Letters above the bars indicated significance groups (*P* < 0.05; one-way ANOVA, Supplemental File S1), lower case letters, wound-induced lesion size; upper case letters, ion leakage. Bar = 0.5 mm. E, *Botrytis-*induced lesion size in the indicated genotype. Bars represent mean ± se (three independent biological replicates; each analyzing leaves from five individual plants; *n* ≥ 53 in total). Letters above the bars indicate significance groups (*P* < 0.05, one-way ANOVA, Supplemental File S1). Bar = 0.5 cm.

T-DNA transformation can result in genome structure changes or have epigenetic impacts, and this may contribute to phenotypes independent of the T-DNA insertion (Jupe et al., 2019). To comprehensively assess the genomic changes in *bos1-1*, Nanopore genome re-sequencing was performed (Brown and Clarke, 2016). This analysis identified 1,173 structural variations in *bos1-1*, including large rearrangements (>1,000 bp), 13 insertions, 16 deletions, 19 duplications, and 24 inversions. In contrast, no mutations were detected in the *BOS1* coding sequence (Supplemental Data Set 2). Because assessing the potential influence of all these changes on *bos1-1* phenotypes is not feasible, we created intragenic double mutant alleles to test the effect of additional exon-disrupting mutations in the *bos1-1* background. Using CRISPR/Cas9, secondary mutations were introduced in exon 2 of *BOS1* in the *bos1-1* mutant background (Figure 4A) resulting in frame shifts (Figure 4B). These new alleles were named bos1-c4* and bos1-c5* (asterisk indicates that the allele is in the *bos1-1* background). These secondary mutations did not attenuate the high *BOS1* transcript level seen in *bos1-1*, as *BOS1* transcript accumulation remained high in both bos1-c4* and bos1-c5* (Figure 4C). The spread of cell death and *Botrytis* susceptibility in bos1-c4* and bos1-c5* were similar to that of wild-type (Figure 4, D and E). The lack of characteristic *bos1-1* phenotypes in these lines indicates that these secondary mutations acted as intragenic suppressors of *bos1-1*. Collectively, these findings demonstrate that the *bos1-1* phenotypes were caused by an alteration of *BOS1* function, rather than other genomic changes.

**Figure 5:**
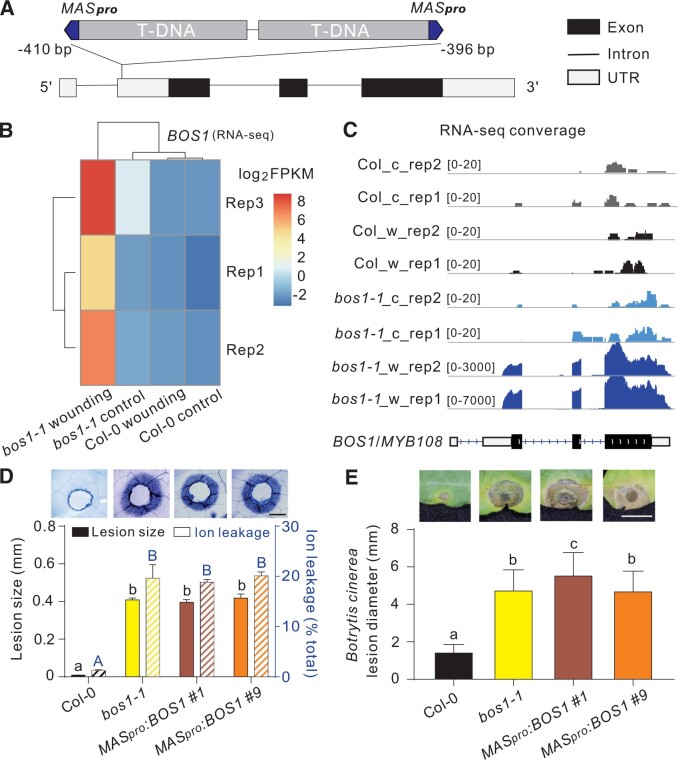
Phenotypes of *bos1-1* are caused by *MAS* promoter-driven *BOS1* expression. A, Schematic illustration of *bos1-1* T-DNA structure. Two adjacent T-DNAs were inserted into the 5′-UTR of *BOS1* with *MAS* promoters indicated in blue. The insertion position of the T-DNAs in *bos1-1* is relative to the *BOS1* start codon. B, Expression of *BOS1* 3 days after wounding. Normalized transcript abundance of *BOS1* was calculated from RNAseq data as fragments per kilobase pair of exon model per million fragments mapped (FPKM). The log2 FPKM values of the indicated genotypes were used to build the heat map. C, RNA-seq reads mapped to *BOS1* genomic DNA. The entire coding sequence of *BOS1* was expressed in *bos1-1*. These data are supported by Supplemental Figures S2 and S8. The c and w indicate control and wounded, respectively. D and E, *MAS_pro_:BOS1* lines phenocopied *bos1-1* upon wounding (D) and *Botrytis* infection (E). D, Representative photos of the spread of cell death from toothpick-puncture wounds, visualized with trypan blue staining and quantified by two methods; measurement of the distance from the wound edge to the outer border of the spreading cell death (black bars; three biological repeats; *n* = 12 in total) and ion leakage of leaves (blue striped bars; three biological repeats; *n* = 12 in total). Letters above the bars indicate significance groups (*P* < 0.05; one-way ANOVA, Supplemental File S1), lower case letters, wound-induced lesion size; upper case letters, ion leakage. Bar = 0.5 mm. This panel is supported by Supplemental Figure S3. E, *Botrytis*-induced lesion size is shown both in representative photos and as quantitative data. Bars represent means ± se (three independent biological replicates; *n* ≥ 45 in total). Letters above the bars indicate significance groups (*P* < 0.05, one-way ANOVA, Supplemental File S1). Bar = 0.5 cm.

We hypothesize that the T-DNA insertion caused an elevated *BOS1* transcript level, which conferred the cell death phenotype in *bos1-1*. The exact site of the T-DNA insertion remains unclear (Kraepiel et al., 2011). Genome resequencing and Sanger sequencing data identified two adjacent T-DNAs in opposite orientations between –410 and –396 bp in the 5′-UTR of *BOS1* (Figure 5A). As expected (Sessions et al., 2002), a *mannopine synthase* (*MAS*) promoter was found adjacent to the left border of each T-DNA (Figure 5A). The *MAS* promoter (*MAS_pro_*) is wounding-inducible and controls gene expression in a bidirectional manner (Guevara-García et al., 1999). Accordingly, the *BOS1* transcript level in *bos1-1* was highly responsive to wounding (Figure 5B). RNA-sequencing (RNA-seq) detected two transcripts, one on each side of the *MAS* promoter; the *BOS1* mRNA with a truncated 5′-UTR at the right flank, and a *BlpR* Basta resistance gene derived from the T-DNA at the left flank (Supplemental Figure S2). The full *BOS1* coding sequence was expressed with no alternative splicing or mutations detected (Figure 5C; Supplemental Figure S2).

These findings suggest that *bos1-1* phenotypes result from high-level *BOS1* transcript accumulation driven by the *MAS_pro_*. To test this, a *MAS_pro_*:*BOS1* construct was transformed into wild-type plants. During generation of this tool, many lines harboring *MAS_pro_*:*BOS1* exhibited enhanced disease susceptibility phenotypes under standard greenhouse conditions and died after flowering (Supplemental Figure S3). This was consistent with our previous observation that *bos1-1* did not survive under greenhouse conditions (Cui et al., 2019). In clean growth room experiments, *MAS_pro_*:*BOS1* lines exhibited spreading cell death upon wounding, and enhanced *Botrytis* susceptibility, similar to *bos1-1* (Figure 5, D and E). Thus, both of the two key *bos1-1* phenotypes were reproduced by introduction of *MAS_pro_*:*BOS1* into wild-type. Overall, we conclude that *bos1-1* is a gain-of-function mutant caused by *MAS_pro_*-driven expression of *BOS1*.

**Figure 6:**
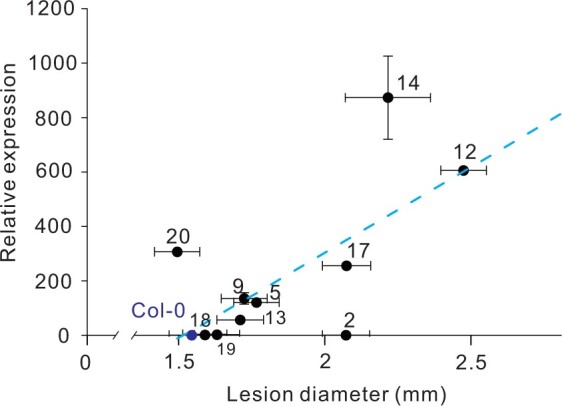
*BOS1* transcript levels were positively correlated with *Botrytis* susceptibility. Relative *BOS1* expression in 11 independent T_3_  *35S_pro_:BOS1* overexpression lines was examined by qPCR. Lesion size was measured as described above. Three independent biological replicates (each replicate consists of leaves from five individual plants; *n* ≥ 48 in total) were combined and analyzed. The blue dashed line indicates the correlation trend. The Pearson coefficient (*r*) of 0.72 for these data indicates a strong correlation between *BOS1* transcript levels and lesion size. The raw data for this figure are available in Supplemental Data Set 1.

Multiple lines of evidence support a connection between *BOS1* transcript level and *bos1-1* phenotypes. Extraordinarily high *BOS1* transcript levels were detected in *bos1-1* upon wounding and infection with *Botrytis* (Figure 4C; Mengiste et al., 2003). Additionally, in genetic experiments, the extent of PCD propagation in *bos1-1* was positively correlated with the *BOS1* transcript level, as *bos1-1/BOS1* had a lower *BOS1* transcript level and proportionally less cell death than did *bos1-1/bos1-1* (Figures 1, C, D and 4, C). To further test this, a series of lines overexpressing *BOS1* under the control of the cauliflower mosaic virus *35S* promoter (*35S_pro_*) were constructed and challenged with *Botrytis*, and this revealed a positive correlation between lesion size and *BOS1* transcript level (Figure 6; Supplemental Data Set 1). This further supports that *bos1-1 Botrytis* susceptibility was conferred by enhanced *BOS1* transcript accumulation.

### BOS1 functions in hormone and abiotic stress responses

The *BOS1* transcript level was elevated in response to multiple stresses (Supplemental Figure S4). In order to assess the role of BOS1 in abiotic stress and hormone responses, the responses to abscisic acid (ABA), methyl viologen, and NaCl were assayed in the new loss-of-function CRISPR/Cas9-induced alleles (Supplemental Figures S5–S7). These experiments revealed increased sensitivity to ABA in these alleles (Supplemental Figure S5). However, mutant responses were indistinguishable from wild-type under stresses induced by NaCl or methyl viologen (Supplemental Figures S6 and S7).

## Discussion

### Is *bos1-1* a loss-of-function or gain-of-function allele?

Previous studies have noted inconsistent phenotypes of *bos1-1* and other *bos1* alleles. For example, *bos1-1* exhibited normal fertility, whereas clearly reduced fertility was observed in alleles with T-DNA insertions in introns (Mandaokar and Browse, 2009). Conversely, *bos1* intronic T-DNA alleles had no pathogen-related phenotypes (Mandaokar and Browse, 2009; Kraepiel et al., 2011). This discrepancy is perhaps related to the interpretation of *bos1-1* as a loss-of-function mutant, which may have resulted from the technological limitations of the time and a lack of other *bos1* alleles available for confirmation of phenotypes. We demonstrate here that cell death spread and *Botrytis* susceptibility in *bos1-1* were the result of a mis-regulated *BOS1* transcript level, rather than a loss of *BOS1* function. However, it is important to note that the publication originally describing *bos1-1* (Mengiste et al., 2003) has several lines of evidence that convincingly demonstrate *bos1-1* was a recessive loss-of-function mutant. This included genetic segregation and genomic complementation analyses. Importantly, the differences in procedures and conditions used in different labs may have altered the phenotypes observed. Differences in growth conditions or infection protocols can significantly influence the extent of *Botrytis* infection (Harper et al., 1981; Ciliberti et al., 2015).

The key differences between our study and that of Mengiste et al. (2003) include the fungal cultivation medium used (2 × V8 versus potato dextrose broth), infection medium used (Sabouraud maltose broth versus potato dextrose broth), and the age of infected plants (3 weeks versus 24 days). These differences may to some extent account for the different results seen between these studies.

Mengiste et al. (2003) present transgenic mutant complementation data. Because relative lesion size in the complemented mutant (*bos1-1* *+* *BOS1*) versus *bos1-1* is much greater in comparison to that in the complemented mutant versus wild-type, Mengiste et al. (2003) evaluate the complementation line as wild-type. However, examination of the original figure reveals that the complemented mutant line exhibited stronger *Botrytis* symptoms than the wild-type, with larger lesion size and enhanced cell death around the lesion frontiers. The choice of the particular *Botrytis* strain may also impact lesion size. This is elegantly illustrated by a study of 96 diverse *Botrytis* isolates that result in contrasting symptoms (Zhang et al., 2017). This study used the Bo5.10 strain, but the exact *Botrytis* strain used by Mengiste et al. (2003) is not specified. We speculate that the fungal cultivation or infection method in might have increased the contrast between disease symptoms of *bos1-1* and heterozygous or complementation lines. This may have obscured the intermediate phenotypes of the heterozygote or complemented line (Mengiste et al., 2003).

### An increased *BOS1* transcript level confers *Botrytis* susceptibility and uncontained cell death

Both *MAS_pro_*- and *35S_pro_*-driven expression of *BOS1* conferred *Botrytis* susceptibility, but with some informative differences. While *MAS_pro_* gave robust phenotypes, use of *35S_pro_* resulted in outcomes that were more variable (Figure 6). *MAS_pro_* conferred strong wound inducible *BOS1* expression (Figure 5, B and C), and this might lead to more precise *BOS1* expression in its target tissue (infection or wound sites) as compared to the general expression patterns of *35S_pro_*. The use of *35S_pro_* can also have unintended consequences. Multiple studies illustrate gene silencing and integration site effects with genes overexpressed using the 35S promoter (Schubert et al., 2004; Daxinger et al., 2008; Mlotshwa et al., 2010; Gelvin, 2017). To further address this, 11 *35S_pro_:BOS1* lines were examined with *Botrytis* infection (Figure 6). Most, but not all, of these overexpression lines exhibited enhanced susceptibility to *Botrytis*. A previous study showed that overexpression of *35S_pro_:BOS1-GUS* increased *Botrytis* resistance (Luo et al., 2010). It cannot be excluded that fusion to *GUS* may have altered *BOS1* function. However, it is common to have some overexpression lines that exhibit different or even opposite phenotypes. In our study, there were two such exceptional lines, #*2* and *#20*, among our 11 *35S_pro_:BOS1* lines (Figure 6). Remarkably, the *BOS1* transcript level in line *#20* was elevated more than 300-fold, but its *Botrytis*-lesion size was slightly reduced (Figure 6). This demonstrates the importance of evaluating many independent overexpression lines for gene function analysis.

### BOS1 also has a function in other biological processes

Under control conditions, *BOS1/MYB108* is mostly expressed in the cell types responsible for anther dehiscence (Mandaokar and Browse, 2009; Xu et al., 2019), and dehiscence requires properly timed PCD for pollen release (Beals and Goldberg, 1997; Senatore et al., 2009; Wilson et al., 2011). Our CRISPR/Cas9-induced loss-of-function alleles exhibited alterations in the extent or timing of dehiscence, similar to *bos1* intronic T-DNA alleles (Figure 3B; Mandaokar and Browse, 2009; Xu et al., 2019). This suggests that *BOS1* could be required for cell death regulation in septum or stomium cells of the dehiscence zone.

Another important regulator of anther development is jasmonic acid (JA; Ishiguro et al., 2001). A characteristic phenotype of JA biosynthesis mutants and strong mutant alleles of the JA receptor CORONATINE INSENSITIVE 1 (COI1) is male sterility (Park et al., 2002; Mandaokar et al., 2003; Jewell and Browse, 2016). Two other MYB transcription factors, MYB21 and MYB24, act downstream from COI1 to regulate anther dehiscence (Song et al., 2011), and MYB24 acts redundantly with MYB108/BOS1 (Mandaokar and Browse, 2009). *BOS1* expression is JA dependent in flowers (Mandaokar and Browse, 2009), thus it is clear that multiple MYB transcription factors coordinate anther development. JA is also crucial for defense against insect and necrotrophic pathogen attack, and a characteristic phenotype for JA deficient or insensitive mutants is of enhanced *Botrytis*-induced lesion size (Thomma et al., 1998; Zhang et al., 2017). *Botrytis* infection leads to elevated *BOS1* transcript levels, and this is dependent on JA signaling via COI1 (Mengiste et al., 2003). These similarities between JA regulation of MYB transcription factors in both anthers and in *Botrytis* infection suggest that common components may be utilized for PCD regulation.

In addition to altered flower development, we show that the *bos1* CRISPR/Cas9-induced alleles exhibited enhanced sensitivity to ABA (Supplemental Figure S5). *BOS1* transcript level was elevated during multiple abiotic and biotic stresses (Supplemental Figure S4). As multiple MYB transcription factors are required for anther development (Mandaokar and Browse, 2009; Song et al., 2011), we propose that stress responses also require the function of several MYB transcription factors.

There are two possible interpretations of the role of BOS1 in stress responses in leaves. Ectopic overexpression of *BOS1* driven by *MAS_pro_* may result in a leaf phenotype that is purely an artifact, suggesting that BOS1 is relevant only in the context of the flower, where it is naturally highly expressed. This argues against a role of BOS1 in stress-induced cell death in leaves and is supported by the lack of leaf cell death and stress phenotypes in *bos1* loss-of-function mutants. Alternatively, the ABA sensitivity phenotype of CRISPR/Cas9-induced *bos1* alleles suggests a role for BOS1 in regulating hormone responses in tissues outside the flower. Support for this is that *BOS1* is stress inducible in leaves (Supplemental Figure S4). Additionally, leaves in the *bos1-1* mutant and *MAS_pro_:BOS1* lines with elevated *BOS1* transcript levels can execute cell death (Figures 1, 4, and 5), which implies the cell death signaling pathway(s) downstream of *BOS1* are active in leaves. Considering these together, the possible relevance of BOS1 to *Botrytis* and other stress responses in the leaf cannot be excluded, and further studies will be required to resolve this question.

### Caution should be observed when using T-DNA lines containing *MAS_pro_*

The original mutant pool used for *bos1-1* isolation was described in McElver et al. (2001) and used the T-DNA vectors pCSA104, pDAP101, and pCSA110. Subsequently, these T-DNA mutant pools were used to create the SAIL collection of indexed T-DNA mutant lines (Sessions et al., 2002). The sequence for these vectors (download: http://seedgenes.org/FlankingSequence.html) were examined, revealing that all three vectors contain the *MAS* promoter (Supplemental Figure S8). *MAS_pro_* is used in these constructs to drive the expression of the bialophos/phosphinothricin (BASTA) resistance gene, *BlpR*. However, the bi-directional promoter activity of *MAS_pro_* may result in enhanced expression of genes adjacent to the T-DNA insertion site (Guevara-García et al., 1999). SAIL lines are an extensively used resource with seeds widely distributed by community stock centers. The presence of a *MAS* promoter in T-DNA insertions of SAIL lines, or possibly other T-DNA mutant collections, may cause unexpected changes in gene expression. Along with the unclear genetic background of the SAIL lines (Nikonorova et al., 2018), caution is advised in the interpretation of results obtained using these mutants. As other vectors for T-DNA transformation also use *MAS_pro_* (Lampropoulos et al., 2013), further critical examination is warranted in the design of experiments that rely on T-DNA insertion mutants.

### Summary

It appears that a re-evaluation of previous generations of genetic tools is required (Nikonorova et al., 2018) because the development of gene editing technologies allows a more accurate examination of gene function. These new tools facilitate re-evaluation of mutants and a refinement of our interpretation of the scientific literature (Westphal et al., 2008; Gao et al., 2015). Here, we have built upon the work of Mengiste et al. (2003) and demonstrated the function of BOS1 as a positive regulator of cell death. Aside from our proposed changes to some interpretations, the majority of Mengiste et al. (2003) remains valid. Based on our previous publications and results presented here, we propose that BOS1 regulates cell death propagation signals from dying cells to neighbor cells, rather than cell death initiation. This role may be of wider interest to the plant research community and warrants further investigation.

## Materials and methods

### Cultivation conditions

Arabidopsis seedlings (Col-0) were transplanted to a 1:1 mixture of peat and vermiculite after 1 week in vitro growth on 1/2 MS medium (Murashige and Skoog, 1962). Plant growth conditions were 23°C/18°C (day/night) temperature, 120–150 μmol m^−2^ s^−2^ light intensity, 12-h/12-h (light/dark) photoperiod, and 60% humidity. *Botrytis* strain BO5.10 obtained from Prof. Jean-Pierre Métraux’s Lab was cultivated on commercial potato dextrose agar (PDA) medium (P2182, Sigma-Aldrich, St. Louis, MO, USA). *Botrytis* plates were kept in the dark at room temperature and transferred into 4°C after conidia production.

### Infection and wounding assays

Fresh *Botrytis* conidia were collected with mycelium into 1/3 strength PDA (P2182, Sigma-Aldrich). The mixture was vortexed and filtered to remove mycelia. Conidia were suspended at 2 × 10^5^ spores mL^−1^. Fully expanded leaves of 24-day-old plants were inoculated with 3 μL of conidial suspension. Plants were covered with a transparent plastic lid to maintain 100% humidity. Symptoms were photographed at 3 days postinoculation (dpi). Wounding was conducted with a toothpick by puncturing fully expanded leaves of 23-day-old plants. Wounding-induced cell death was visualized by trypan blue staining, with wounded leaves collected at 4-day postwounding (dpw). Both *Botrytis* lesion size and wounding-induced cell death were measured from photographs using ImageJ (http://rsb.info.nih.gov/ij/). Procedures for cell death staining were described previously (Cui et al., 2013, 2019). For ion leakage measurements, three to four leaves were wounded with a bundle of toothpicks resulting in a matrix of wounds each 2-mm apart. At 5 dpw, leaves were submerged in ultrapure milliQ water, and resulting ion leakage was measured 4 h later with a conductivity meter as described in Cui et al. (2018). Ion leakage was expressed as the percentage of total ions that remained after disrupting all membranes by freezing the sample leaves.

### Seedling growth assays

For ABA and NaCl treatments, sterilized seeds were sown on 1/2 MS media containing ABA or NaCl at the indicated concentrations. Root lengths were photographed at 9 days after sowing and were measured using ImageJ (http://rsb.info.nih.gov/ij/). For methyl viologen treatment, seeds were germinated on control plates and 4-day-old seedlings were transferred to media with the indicated concentrations of methyl viologen. Photos were taken at 15 days after transplanting.

### Cloning procedures

*BOS1* genomic DNA was cloned into the vector pGWB412 (Addgene plasmid # 74806) to construct the *35S_pro_:BOS1* plasmid. The *MAS_pro_* was amplified using template DNA from the *bos1-1* mutant, and then used to replace the 35S promoter of *35S_pro_:BOS1* to create *MAS_pro_:BOS1*. To create the new CRISPR/Cas9-induced loss-of-function alleles, guide RNA (gRNA) targeting the first and second exons of *BOS1* were integrated into pCBC_DT1DT2 and then into the final vector pHEC401 according to (Xing et al., 2014). Vectors were transformed into the indicated plants via Agrobacterium strain GV3101 (TSINGKE #TSC-A01, China), by the ﬂoral dip method (Clough and Bent, 1998). The primers used in this study are listed in Supplemental Table S1.

### Transformation procedures

The *bos1-1* mutant was found to be incompatible with *Agrobacterium* transformation. Test transformations of *bos1-1* were performed in labs in Helsinki, Finland and Hangzhou, China. All transformed *bos1-1* plants died before seed set due to spreading cell death trigged by *Agrobacterium*. To overcome this limitation, CRISPR/Cas9 vectors were first transformed into wild-type plants, with integration presumably into a chromosomal location not linked with *BOS1*. The vector was then transferred into the *bos1-1* background by crossing using a CRISPR/Cas9 vector transformant as a pollen donor and *bos1-1* as the pollen acceptor. The vectors functioned in the F_1_ generation to modify the BOS1 locus, and then were removed by segregation in the F_2_. Homozyotes of *bos1-1* were selected by PCR and then screened for bos1-c4* and -c5* by Sanger sequencing.

### Genome re-sequencing

Genomic DNA of *bos1-1* was extracted and sequenced by the Biomarker Technologies Corporation (Beijing, China) following the standard procedures of Oxford Nanopore Technology sequencing (Deamer et al., 2016). Sequence depth was 129×, 99.77% of 24.37 GB clean data mapped properly to the Arabidopsis genome (TAIR10). The raw data have been deposited to NCBI (PRJNA728243). Structural variations were analyzed with Sniffles (Sedlazeck et al., 2018).

### RNA-seq

Fully expanded leaves of 23-day-old plants were punctured with a bundle of toothpicks, and collected after 3 days. Unwounded plants were used as a control. RNA was extracted using the TRIzol reagent (Invitrogen, Carlsbad, CA, USA). Library construction and sequencing were carried out in LC-BIO Biotech Ltd with Illumina Hiseq 4000. Raw reads were filtered and aligned to the Arabidopsis genome (TAIR10) using hisat2 (version 2.1.0; Kim et al., 2015). To identify the transcripts adjacent to *MAS_pro_*, the conjoined sequence of the T-DNA and *BOS1* genome sequence were first obtained from the *bos1-1* mutant resequencing analysis, and their sequences verified with Sanger sequencing. Then the combined sequence was used as a reference for read mapping. The RNA-seq raw data have been deposited to NCBI (PRJNA728243). Normalized transcript abundances of *BOS1* were calculated as fragments per kilobase pair of exon model per million fragments mapped with Cufflinks (Trapnell et al., 2010). For real-time quantitative PCR (qPCR), leaves of 23-day-old plants were used for RNA extraction and reverse transcription. The raw cycle threshold values were analyzed with Qbase+ (Biogazelle; Hellemans et al., 2007) with the reference genes *ACTIN2*, *PP2AA3*, and *ACTIN8*.

### Statistical analysis

One-way ANOVA (Analysis of Variance) and *t* tests were used for statistical analysis as indicated in figure legends. One-way ANOVA analysis was performed using Graphpad Prism version 7 (Mitteer et al., 2018). Briefly, all data of different biological repeats were statistically analyzed using Tukey’s multiple comparisons test with default setting. T tests were performed in Microsoft Excel 2016 using two-tailed and unpaired settings. Statistical analysis tables are presented in Supplemental File S1.

## Accession numbers

Gene identifiers for Arabidopsis are *BOS1/MYB108* (AT3G06490), *ACTIN2* (AT3G18780), *PP2AA3* (AT1G13320), and *ACTIN8* (AT1G49240). New sequencing data, including *bos1-1* resequencing data and RNA-seq data can be found at the NCBI SRA (PRJNA728243).

## Supplemental data

The following materials are available in the online version of this article.

**Supplemental Figure S1.** Alignments of protein sequences of BOS1/MYB108 and the truncated proteins of the new *bos1* alleles.

**Supplemental Figure S2.** Sequences and positions of the transcripts at the *BOS1* locus in the *bos1-1* mutant.

**Supplemental Figure S3.** Col-0 lines transgenically expressing *MAS_pro_:BOS1* exhibited enhanced disease susceptibility under standard greenhouse conditions.

**Supplemental Figure S4.**  *BOS1* transcript levels in Arabidopsis RNAseq data conditions outputed from the Genevestigator (Hruz et al., 2008).

**Supplemental Figure S5.** The CRISPR/Cas9-induced *bos1* loss-of-function alleles showed enhanced ABA sensitivity.

**Supplemental Figure S6.** The CRISPR/Cas9-induced *bos1* loss-of-function alleles showed a wild-type methyl viologen (MV) response.

**Supplemental Figure S7.** The CRISPR/Cas9-induced *bos1* loss-of-function alleles exhibited unaltered NaCl sensitivity.

**Supplemental Figure S8.** Architecture of the T-DNA insertion in *bos1*-*1.*

**Supplemental Table S1.** Primers used in this study.

**Supplemental Data Set 1.** Raw data for Figure 6, including lesion sizes and *BOS1* transcript levels.

**Supplemental Data Set 2.** Identification of genomic changes in *bos1-1* that were identified by genome re-sequencing.

**Supplemental File S1.** Statistical analysis tables.

## Supplementary Material

koac206_Supplementary_Data

## Notes

F.C., K.O., and M.B. designed the project. X.L., W.L., and Y.W. performed the experiments. W.W. conduced the bioinformatic analyses. F.C. and X.L. wrote the first draft of the manuscript. All authors edited, proofread, and approved the manuscript. F.C. obtained financial support.

The author responsible for distribution of materials integral to the findings presented in this article in accordance with the policy described in the Instructions for Authors (https://academic.oup.com/plcell) is: Fuqiang Cui (fuqiang.cui@zafu.edu.cn).
