# Supplementary material for: Ectopic expression of BOTRYTIS SUSCEPTIBLE1 reveals its function as a positive regulator of wound-induced cell death and plant susceptibility to Botrytis
Source: Plant Cell. 2022 Aug 10;34(10):4105–16. doi: 10.1093/plcell/koac206 (PMC9516177; doi:10.1093/plcell/koac206)
Supplement: koac206_Supplementary_Data [file koac206_supplementary_data.zip › koac206_Supplementary_Data/tpc.22.00079_SupplementalFiguresandTables.pdf]

```
                      ******************************************
BOS1/MYB108           MDEKGRSLKNNNMEDEMDLKRGPWTAEEDFKLMNYIATNGEGRWNSLSRCAGLQRTGKSC     60
bos1-c1               MDEKGRSLKNNNMEDEMDLKRGPWTAEEDFKLMNYIATNGEGALELSFSLRRPPTHR*      57
bos1-c2               MDEKGRSLKNNNMEDEMDLKRGPWTAEEDFKLMNYIATNGEGRWNSLSRCAGLQRTGKSC     60
bos1-c3               MDEKGRSLKNNNMEDEMDLKRGPWTAEEDFKLMNYIATNGEGRWNSLSRCAGLQRTGKSC     60
bos1-c4*              MDEKGRSLKNNNMEDEMDLKRGPWTAEEDFKLMNYIATNGEGRWNSLSRCAGLQRTGKSC     60
bos1-c5*_var1         MDEKGRSLKNNNMEDEMDLKRGPWTAEEDFKLMNYIATNGEGRWNSLSRCAGLQRTGKSC     60
bos1-c5*_var2         MDEKGRSLKNNNMEDEMDLKRGPWTAEEDFKLMNYIATNGEGRWNSLSRCAGLQRTGKSC     60

BOS1/MYB108           RLRWLNYLRPDVRRGNITLEEQLLILELHSRWGNRWSKIAQYLPGRTDNEIKNYWRTRVQ     120
bos1-c1               ------------------------------------------------------------     57
bos1-c2               RLRWLNYLRPDVPVETLHLKNNS*------------------------------------     83
bos1-c3               RLRWLNYLRPDVRPWKHYT*----------------------------------------     79
bos1-c4*              RLRWLNYLRPDVRPWKHYT*----------------------------------------     79
bos1-c5*_var1         RLRWLNYLRPDVPVETLHLKNNS*------------------------------------     83
bos1-c5*_var2         RLRWLNYLRPDVRPWKHYT*----------------------------------------     79

BOS1/MYB108           KHAKQLKCDVNSQQFKDTMKYLWMPRLVERIQSASASSAAAATTTTTTTTGSAGTSSCIT     180
bos1-c1               ------------------------------------------------------------     57
bos1-c2               ------------------------------------------------------------     83
bos1-c3               ------------------------------------------------------------     79
bos1-c4*              ------------------------------------------------------------     79
bos1-c5*_var1         ------------------------------------------------------------     83
bos1-c5*_var2         ------------------------------------------------------------     79

BOS1/MYB108           TSNNQFMNYDYNNNNMGQQFGVMSNNDYITPENSSVAVSPASDLTEYYSAPNPNPEYYSG     240
bos1-c1               ------------------------------------------------------------     57
bos1-c2               ------------------------------------------------------------     83
bos1-c3               ------------------------------------------------------------     79
bos1-c4*              ------------------------------------------------------------     79
bos1-c5*_var1         ------------------------------------------------------------     83
bos1-c5*_var2         ------------------------------------------------------------     79

BOS1/MYB108           QMGNSYYPDQNLVSSQLLPDNYFDYSGLLDEDLTAMQEQSNLSWFENINGAASSSDSLWN     300
bos1-c1               ------------------------------------------------------------     57
bos1-c2               ------------------------------------------------------------     83
bos1-c3               ------------------------------------------------------------     79
bos1-c4*              ------------------------------------------------------------     79
bos1-c5*_var1         ------------------------------------------------------------     83
bos1-c5*_var2         ------------------------------------------------------------     79

BOS1/MYB108           IGETDEEFWFLQQQQQFNNNGSF*  323
bos1-c1               ------------------------  57
bos1-c2               ------------------------  83
bos1-c3               ------------------------  79
bos1-c4*              ------------------------  79
bos1-c5*_var1         ------------------------  83
bos1-c5*_var2         ------------------------  79
```

Supplemental Figure S1. Alignments of protein sequences of BOS1/MYB108 and the truncated proteins of the new *bos1* alleles (Supports Figures 1 and 4).

BOS1/MYB108 was aligned with the peptides encoded by the Crispr/Cas9-induced loss-of-function alleles *bos1-c1* to *-c3* (see Figure 1) and *bos1-1* intergenic double mutant alleles *bos1*-c4* and *bos1*-c5* (see Figure 4). Missense sequences induced by mutation are indicated in magenta letters. There were two variants found segregating in the *bos1-c5** allele, which are listed as var1 and var2.

>T-DNA transcript including the BlpR resistance gene
TCAGGACTTTTTTTTTTTTTTTTTTTTTTTTAGGAATTAGAAATTTTATTGATAGAAGTATTTTACAAATACAAATACATAC
TAAGGGTTTCTTATATGCTCAACACATGAGCGAAACCCTATAAGAACCCTAATTCCCTTATCTGGGAACTACTCACACA
TTATTATAGAGAGAGATAGATTTGTAGAGAGAGACTGGTGATTTCAGCGGGCATGCCTGCAGGTCGACTCTAGAGGA
TCCTAGAACGCGTGATCTCAGATCTCGGTGACGGGCAGGACCGGACGGGGCGGTACCGGCAGGCTGAAGTCCAGC
TGCCAGAAACCCACGTCATGCCAGTTCCCGTGCTTGAAGCCGGCCGCCCGCAGCATGCCGCGGGGGGCATATCCG
AGCGCCTCGTGCATGCGCACGCTCGGGTCGTTGGGCAGCCCGATGACAGCGACCACGCTCTTGAAGCCCTGTGCC
TCCAGGGACTTCAGCAGGTGGGTGTAGAGCGTGGAGCCCAGTCCCGTCCGCTGGTGGCGGGGGGAGACGTACACG
GTCGACTCGGCCGTCCAGTCGTAGGCGTTGCGTGCCTTCCAGGGGCCCGCGTAGGCGATGCCGGCGACCTCGCCG
TCCACCTCGGCGACGAGCCAGGGATAGCGCTCCCGCAGACGGACGAGGTCGTCCGTCCACTCCTGCGGTTCCTGC
GGCTCGGTACGGAAGTTGACCGTGCTTGTCTCGATGTAGTGGTTGACGATGGTGCAGACCGCCGGCATGTCCGCCT
CGGTGGCACGGCGGATGTCGGCCGGGCGTCGTTCTGGGCTCATGGATCCACGTGTGGAAGATATGAATTTTTTTGA
GAAACTAGATAAGATTAATGAATATCGGTGTTTTGGTTTTTTCTTGTGGCCGTCTTTGTTTATATTGAGATTTTTCAAAT
CAGTGCGCAAGACGTGACGTAAGTATCCGAGTCAGTTTTTATTTTTCTACTAATTTGGTCGTTTATTTCGGCGTGTAGG
ACATGGCAACCGGGCCTGAATTTCGCGGGTATTCTGTTTCTATTCCAACTTTTTCTTGATCCGCAGCCATTAACGACTT
TTGAATAGATACGCTGACACGCCAAGCCTCGCTAGTCAAAAGTGTACCAAACAACGCTTTACAGCAAGAACGGAATG
CGCGTGACGCTCGCGGTGACGCCATTTCGCCTTTTCAGAAATGGATAAATAGCCTTGCTTCCTATTATATCTTCCCAA
ATTACCAATACATTACACTAGCATCTGAATTTCATAACCAATCTCGATACACCAAATCGAATTCAATTCGGCGTTAATTC
AGTACATTAAAAACGTCCGCAATGTGTTATTAAGTTGTCTAAGCGTCAATTTGTTTACACCACAATATTGTGGACAAAT
TT

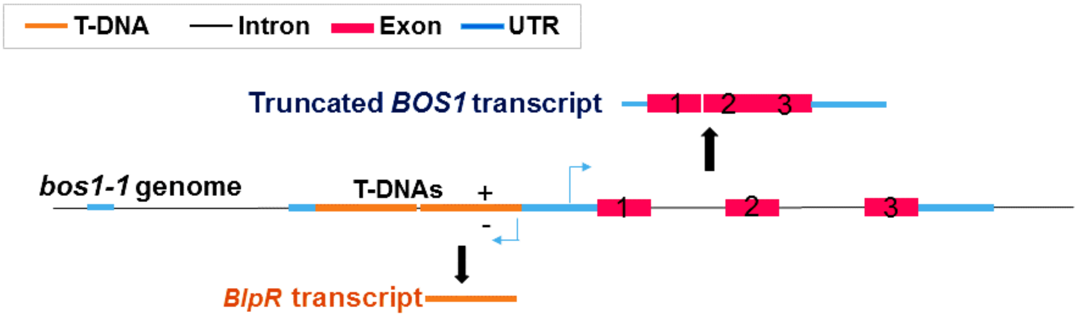

>*BOS1*_transcript
CGGCTGTCTATTCCCTTCATCGCACATGTTCCAAATAATTTTAAAAAATAAAAAGAAAAATTGAAAACTATCTTCTTTTT
TCTTCTATAAAACCCACAACTTCTCTTTTTCTTGTGCATTCAAAACTCATCCTATCTCTATCTACACATAACTCCAAAAAA
CAAACAAATTTTCTCTCTCTATCTCTCTTCGCAAAACAACATAGAAAAAAAGTAGAAAGTCTCAATCTTTTTGCTGAACA
ATCTTGTTGTGGTCTCTTCTGTGTATATCAATGGATGAAAAAGGAAGAAGCTTGAAGAACAACAACATGGAAGACGAG
ATGGACCTAAAGAGAGGTCCGTGGACTGCTGAAGAAGATTTTAAGCTCATGAATTACATTGCTACTAATGGAGAAGGT
CGCTGGAACTCTCTTTCTCGTTGCGCCGGCCTCCAACGCACCGGTAAAAGCTGTAGACTAAGGTGGTTAAACTATCT
CCGCCCTGACGTCCGCCGTGGAAACATTACACTTGAAGAACAACTCTTGATCCTCGAACTTCATTCCCGTTGGGGAA
ATAGATGGTCAAAAATCGCACAATATTTACCGGGAAGAACGGACAACGAGATCAAGAACTACTGGAGGACGCGGGTG
CAAAAGCATGCGAAACAGTTGAAATGTGATGTGAATAGCCAACAATTCAAAGACACAATGAAGTACTTGTGGATGCCT
CGACTAGTCGAGAGGATTCAGTCAGCCTCGGCCTCATCCGCAGCAGCAGCCACCACCACAACCACCACCACCACAG
GATCAGCCGGCACGTCATCTTGCATCACAACCTCTAACAATCAATTCATGAATTACGACTACAACAACAACAACATGG
GACAACAGTTTGGTGTAATGAGCAACAATGATTATATCACGCCTGAAAATTCCAGCGTGGCAGTGTCTCCGGCGTCA
GACTTAACGGAGTACTACAGCGCTCCAAACCCTAACCCGGAATACTATTCGGGTCAAATGGGGAATAGTTATTATCCA
GATCAGAATTTAGTGAGTTCACAATTATTACCGGATAATTATTTCGACTATAGTGGATTATTAGACGAAGATCTAACGG
CTATGCAAGAGCAGAGTAACCTCAGCTGGTTTGAAAACATTAATGGTGCTGCTTCTTCTTCAGACAGTTTATGGAACA
TTGGAGAAACTGATGAAGAATTCTGGTTCTTACAGCAGCAACAACAGTTCAACAATAATGGTAGCTTCTGAAGTTAGA
AAAAAAAAATAGAAATCGTTTTAAGTTAAATTATACACTATAGTATACGTGTGAAAGGAATTTGTTGTAAGGGAATAATT
AAAAACAAAGAATTGTTCATAGGATATATGATCAGGTTTTTTATACCAAGCTTGATCATATATCATGGCGTTTTAACAAA
GCGCTAAACTTGATTGGTTTTGTTTTTTGGGGGGGATTGCAATGATATTTTGTGGATAATTGATAAACTTGGATGAAAT
AATTTATGATTTAATGTTTGGAAAATAACTCATTCGTCGGTGGGACTATGTATACTATAAACATAAATAATGGAAATTGT
ATTCACACTATAGATTGAGTGATTTCCTCAATGGAAGCATAATATAAAACATTATTCATTTAAAAAAAAAAGAA

Supplemental Figure S2. Sequences and positions of the transcripts at the *BOS1* locus in the *bos1-1* mutant (Supports Figure 5).

Two transcripts were found in the RNA-seq analysis. One is the 5'-truncated *BOS1* transcript with a shorter 5'-UTR, and the other is the expected T-DNA transcript including the *BlpR* resistance gene. Blue, untranslated regions; magenta, coding sequence.

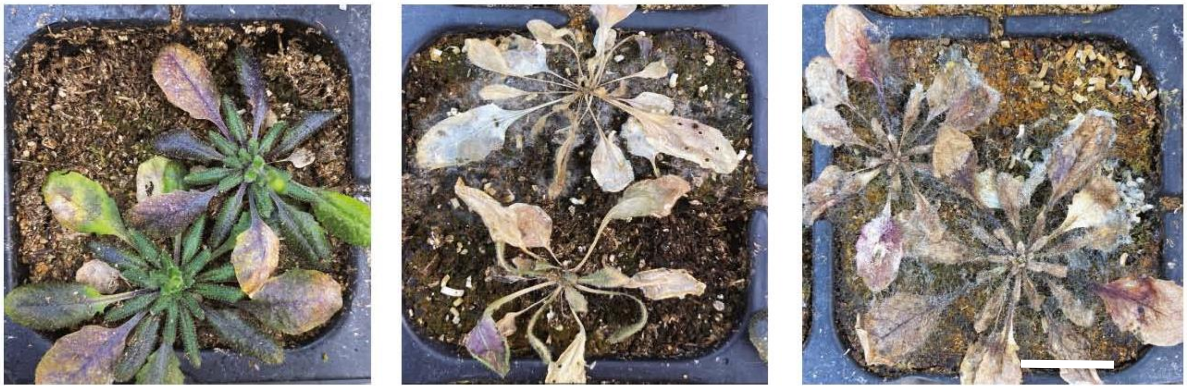

Col-0 *bos1-1* MAS_{pro}*:BOS1*

Supplemental Figure S3. Col-0 lines transgenically expressing MAS_{pro}*:BOS1* exhibited enhanced disease susceptibility under standard greenhouse conditions (Supports Figure 5).

Many MAS_{pro}*:BOS1* lines were infected and died before setting seed. Plants were grown in the greenhouse without fungicide application; representative individuals of the indicated genotypes are shown. Bar = 2 cm.

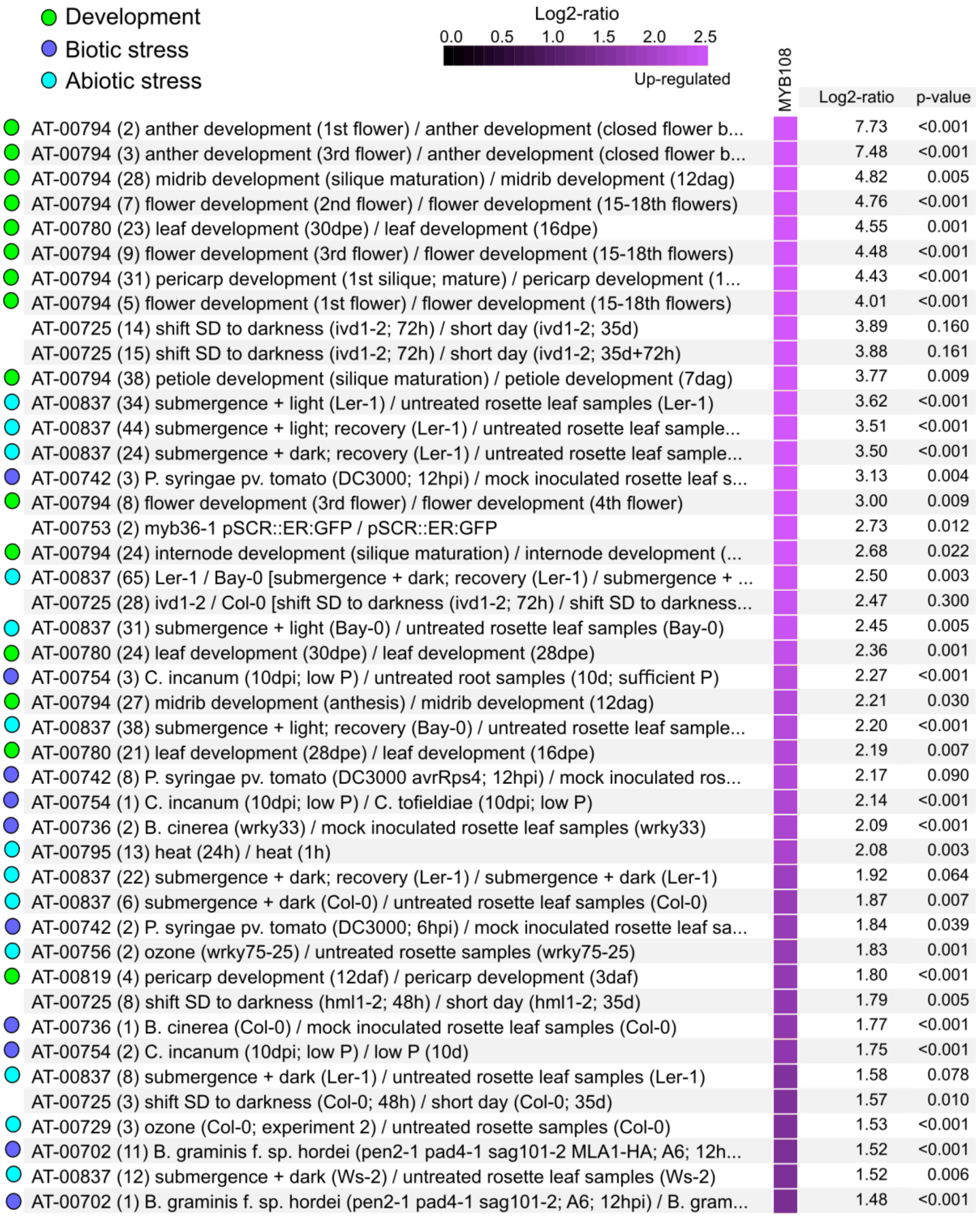

Supplemental Figure S4. *BOS1* transcript levels in Arabidopsis RNAseq data conditions (Supports Figure 3).

The Genevestigator perturbation tool was used to identify experiments with highest up-regulation of *BOS1* transcript level (Hruz et al., 2008). The identification number for each experiment refers to the identifier in the Genevestigator database. The *P*-values were calculated automatically using the export function of the Genevestigator software.

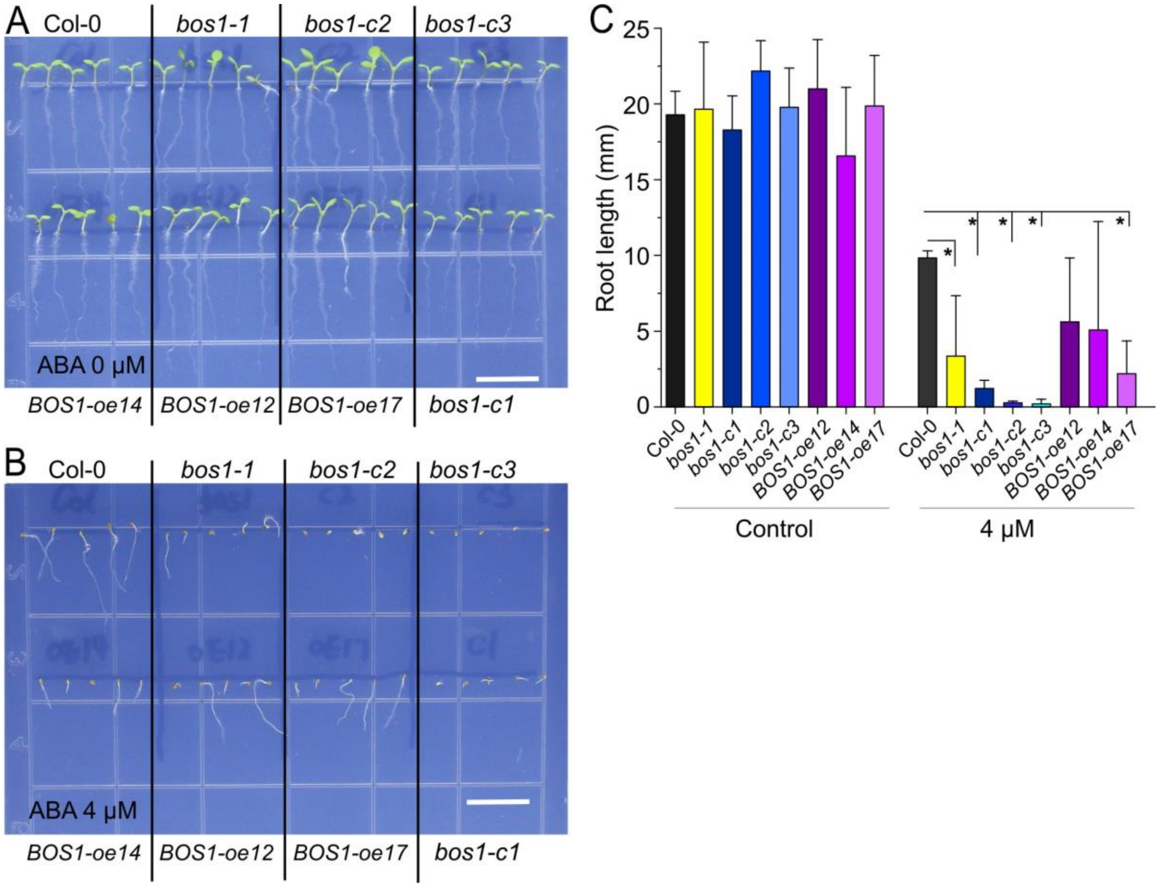

Supplemental Figure S5. The Crispr/Cas9-induced *bos1* loss-of-function alleles showed enhanced ABA sensitivity (Supports Figure 3).

(A) and **(B)** Growth phenotypes in response to 0 μM **(A)** or 4 μM **(B)** ABA. These experiments were repeated twice with similar results. Bar = 1 cm.
(C) Pooled quantitative data of the root lengths from two independent biological repeats (*n* = 10 seedlings total, 5 in each experiment). Bars represent means ± SE, asterisks indicate groups with significantly different root lengths ($P < 0.05$; *t*-test, Supplemental File 1).

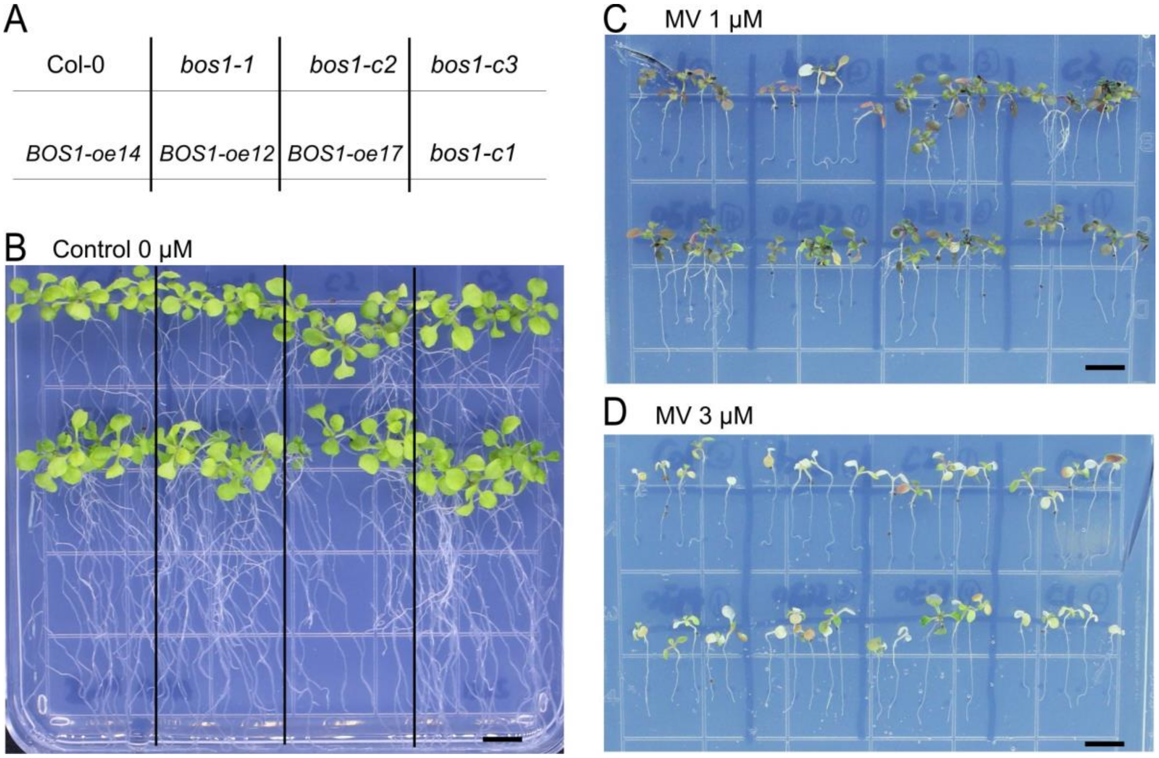

Supplemental Figure S6. The Crispr/Cas9-induced *bos1* loss-of-function alleles showed a wild-type methyl viologen (MV) response (Supports Figure 3).

(A) Layout of the positions of the plant genotypes used in parts **(B)** to **(D).** Symptoms of plants are shown in response to MV at 0 μM **(B)**, 1 μM **(C)**, or 3 μM MV **(D)**. These experiments were repeated twice with similar results and one representative experiment is shown. Bars = 1 cm.

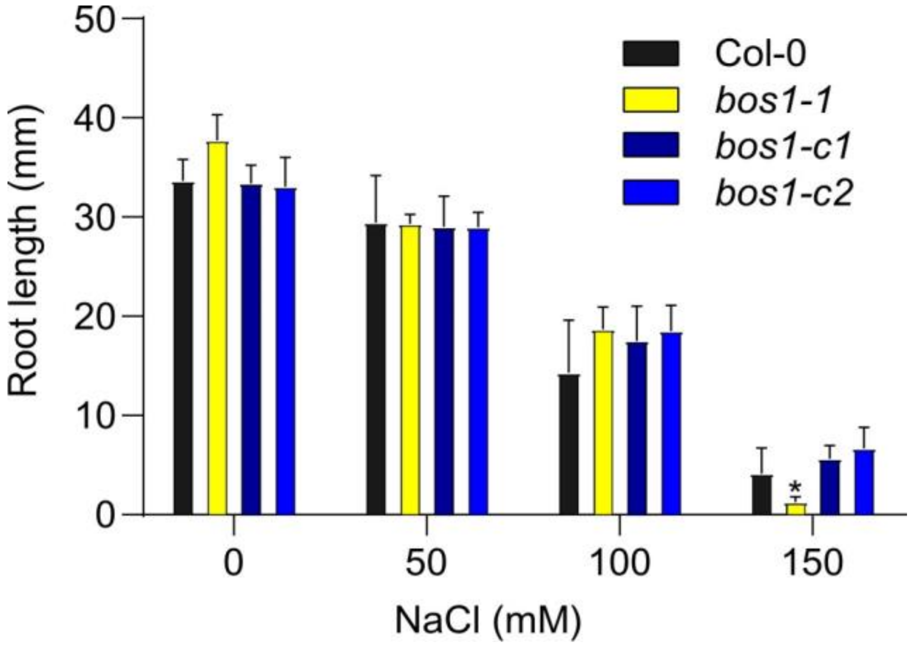

Supplemental Figure S7. The Crispr/Cas9-induced *bos1* loss-of-function alleles exhibited unaltered NaCl sensitivity (Supports Figure 3).

Root lengths of the indicated genotypes were measured on the 9th day. The experiments were repeated twice with similar results (n = 12 roots total, 6 in each experiment). Asterisks indicate groups that have significantly different root lengths ($P < 0.05$; t-test, Supplemental File 1).

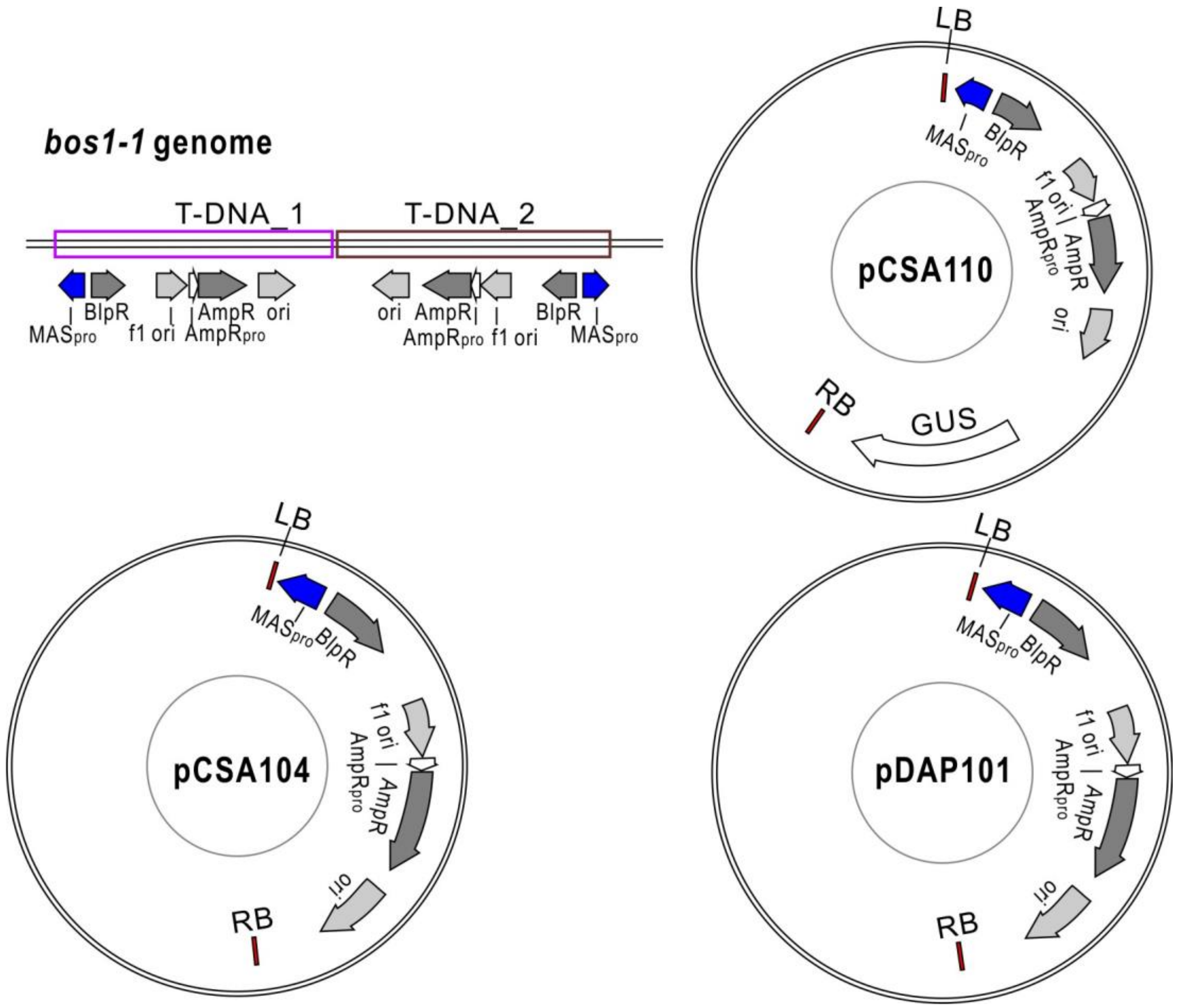

Supplemental Figure S8. Architecture of the T-DNA insertion in *bos1-1* (Supports Figure 5).

The T-DNA insertion in *bos1-1* had a similar architecture and the same *MAS$_{pro}$:BlpR* (BASTA resistance) cassette at the left border as is found in all the three vectors (pCSA104, pDAP101, pCSA110) that were used to construct the SAIL mutant lines of *Arabidopsis*. Vector sequences were obtained from http://seedgenes.org/FlankingSequence.html. The *MAS* promoters are marked with blue arrows.

Supplemental Table S1. Primers used in this study.

Primer names	Sequence (5´ to 3´)
**Primers for vector construction**	
pGWB412-*BOS1*-F	GGGGACAAGTTTGTACAAAAAAGCAGGCTTC ATGGATGAAAAAGGAAGAAGCTTG
pGWB412-*BOS1*-R	GGGGACCACTTTGTACAAGAAAGCTGGGTTTTT CAGAAGCTACCATTATTGTTGAAC
pGWB412-*MAS*-F	CCCAAGCTTGGGTTCAGCAGGTGGGTGTAGAGC
pGWB412-*MAS*-R	GCTCTAGAGCTGATATACACAGAAGAGACCACAACAAG
**Primers for genotyping**	
BOS1T-F	GTCTTAACCTTCACGCACATAAAA
BOS1T-R	CGCAACGAGAAAGAGAGTTCC
BOS1-TDNA-F	GCGTAGTTGCTTTGAGCGTGG
BOS1-TDNA-R	GGCAAACGCTTTACGCTGAAAC
BOS1-E_2E_3-F	GGGAAGAGCACTAACTCAATGG
BOS1-E_2E_3-R	CTGACTGAATCCTCTCGACTAG
BOS1-P_3E_1-F	ATTACGGCTGTCTATTCCCTTC
BOS1-P_3E_1-R	CATACCGGCGCAACGAGAAA
**Primers for quantitative real-time RNA**	
BOS1-qF-1	ACGGCTATGCAAGAGCAGAGTAAC
BOS1-qR-1	ACTGTCTGAAGAAGAAGCAGCAC
AtACTIN2-qF	GGTAACATTGTGCTCAGTGGTGG
AtACTIN2-qR	GACAACCTTAATCTTCATGCTGC
AtACTIN8-qF	ATGACTCAGATCATGTTTGAGACC

AtACTIN8-qR	TCAGTAAGGTCACGACCAGCAA
**Primers for Crispr mutation construction**	
BOS1-Ex1-F0	TGTACTAATGGAGAAGGTCGCGTTTT AGAGCTAGAAATAGC
BOS1-Ex1-BsF	ATATATGGTCTCGATTGTACTAAT GGAGAAGGTCGCGTT
BOS1-Ex2-F0	TGTCCGCCCTGACGTCCGCCGGTTTT AGAGCTAGAAATAGC
BOS1-Ex2-BsF	ATATATGGTCTCGATTGTCCGCCC TGACGTCCGCCGGTT
BOS1-Ex3-R0	AACGCGTCCTCCAGTAGTTCTTCAA TCTCTTAGTCGACTCTAC
BOS1-Ex3-RsR	ATTATTGGTCTCGAAACGCGTCC TCCAGTAGTTCTTC
BOS1-Pr3-R0	AACCAACAAGATTGTTCAGCAAC AATCTCTTAGTCGACTCTAC
BOS1-Pr3-BsR	ATTATTGGTCTCGAAACCAACA AGATTGTTCAGCAAC
