# Supplementary material for: Ectopic expression of BOTRYTIS SUSCEPTIBLE1 reveals its function as a positive regulator of wound-induced cell death and plant susceptibility to Botrytis
Source: Plant Cell. 2022 Aug 10;34(10):4105–16. doi: 10.1093/plcell/koac206 (PMC9516177; doi:10.1093/plcell/koac206)
Supplement: koac206_Supplementary_Data [file tpc.22.00079.PeerReviewReport.pdf › koac206_Supplementary_Data/tpc.22.00079.PeerReviewReport.pdf]

THE PLANT CELL Peer Review Report

Ectopic expression of BOTRYTIS SUSCEPTIBLE1 reveals its function as a positive regulator of wound-induced cell death and plant susceptibility to *Botrytis*.

Fuqiang Cui, Xiaoxiao Li, Wenwu Wu, Wenbo Luo, Ying Wu, Mikael Brosché, and Kirk Overmyer

Corresponding author: Fuqiang Cui, fuqiang.cui@gmail.com

Review timeline:

**TPC2021-LTE-00447**	Submission received:	May 28, 2021	
	1st Decision:	June 3, 2021	*manuscript declined*
**TPC2021-RA-00979D**	Submission received:	Nov. 10, 2021	
	1st Decision:	Nov. 22, 2021	*manuscript declined*
**TPC2022-RA-00079D**	Submission received:	Jan. 20, 2022	
	1st Decision:	March 11, 2022	*revision requested*
**TPC2022-RA-00079DR1**	1st Revision received:	May 11, 2022	
	2nd Decision:	May 31, 2022	*acceptance pending, sent to science editor*
	Final acceptance:	July 13, 2022	

This report shows the major requests for revision and author responses. Minor comments for revision and miscellaneous correspondence are not included. The original format may not be reflected in this compilation, but the reviewer comments and author responses are not edited, except to correct minor typographical or spelling errors that could be a source of ambiguity.

TPC2021-LTE-00447 1st Editorial decision – *declined without external review* June 3, 2021

Thank you for submitting your manuscript entitled "BOS1 is a positive regulator of wounding induced cell death and plant susceptibility to Botrytis" to The Plant Cell. Your submission has been evaluated by members of the editorial board, and we regret to inform you that we are not recommending that your manuscript in its current form proceed further in the review process.

We feel it best to not send your paper out for review until you have had sufficient time to complete this work. We have not made this decision lightly. Your submission was assessed at this stage by three editorial board members who judged that the work would not be appropriate for The Plant Cell, and initiating additional review would only delay the eventual publication of your story. This decision reflects the priorities and platform of The Plant Cell, and is not meant to indicate that the manuscript is unsuitable for publication elsewhere.

The editors judged that your manuscript, in its current form, does not represent a sufficient advance to warrant further consideration at TPC. This letter has some problems that would need to be addressed before we can consider it for external review.

> **RESPONSE TO EDITORS: We thank the editors for their time in evaluating our manuscript. We have performed the requested new experiments, and expanded our manuscript to the full research report format. This allowed us to properly discuss our results in the context of previous knowledge about the bos1 mutants, including the important first publication by Mengiste et al., 2003. As requested, we have also performed new experiments with all of our new lines in response to Botrytis infection, to allow comparisons with the original publication.**

Point 1. The three CRISPR mutants appear to have no phenotype on their own. The strongest evidence is the double mutant incorporating the exon mutants in the T-DNA lineage; this would suggest that there is gene redundancy that is being overlooked.

> **RESPONSE: The loss-of-function mutants of BOS1 created with Crispr-cas9 had no phenotypes in response to wounding or Botrytis infection. However, we have additional data on the fertility phenotypes of these alleles, which was left out of the original submission due to space constraints. The new bos1 crispr alleles, but not bos1-1, exhibited deficiencies in fertility (see Fig. 2, in the revised manuscript), which is consistent with the previous study**

conducted by Mandaokar and Browse, 2009, which used bos1 T-DNA insertion alleles. For details of the fertility phenotypes of bos1 Crispr-Cas9 lines, please see Figure 2 and lines 135-147 of the text.

Point 2. The editors judge that the letter is misleading in suggesting or implying that the original Mengiste et al. (2003) paper is "wrong." The letter suggests that the function of BOS1 is positive rather than negative - but the underlying function is still the same. The original researchers were limited to the technologies available at the time and possibly were mistaken about the directionality.

RESPONSE: We can honestly state it was never our intention to misrepresent, negate, or neglect the findings of Mengiste et al, 2003 paper. Although rereading our own manuscript while taking your comments into account, we agree there is the appearance of such. Many aspects relevant to this manuscript, including the Mengiste et al, 2003 paper, were not properly discussed in an effort to keep the manuscript within the limited space of the letter format. Now that we have expanded the manuscript to the full research report format, we have given the Mengiste et al paper the attention that it deserves. Beyond the space issues, we also acknowledge that previously we had not exercised enough care in presenting our own work in a way that highlights the many positive aspects of the Mengiste et al., 2003 paper. We have always held this paper in high regard and our work only builds upon their findings, which is now reflected in our revised manuscript. Overall, the new contribution in our manuscript is to propose that BOS1 is a positive, rather than negative, regulator of Botrytis susceptibility and cell death.

Point 3. The letter is furthermore a bit misleading about the dominant/recessive topic. In the original paper the data clearly show that the original bos1 mutation behaves as a recessive phenotype; there is an entire section on recessive vs. dominance in the original paper and it is solely about the Botrytis assay, and shows that in that case, the overly sensitive phenotype was recessive with respect to Botrytis infection. It also showed that the phenotype could be complemented by introduction of a wild-type genomic copy of the BOS1 gene, which your letter does not address. Those two pieces of data together are fully consistent with the original mutation being a loss-of-function, thus as stated above, there is nothing that needs to be "corrected" in the original paper. The editors felt that your letter misrepresents and downplays that earlier data by merely saying "The bos1-1 allele was considered recessive".

RESPONSE: As stated above, it was never our intention to misrepresent and downplay the results of Mengiste et al., 2003. Please accept our apologies for not crafting our previous submission with greater care. Also, please see also the answer to point 2 above. In response to the issues of the recessive trait and complementation data-- we now discuss possible reasons that may account for the differences in results and interpretation supporting different conclusions about the genetics of BOS1 and the directionality of BOS1 in Botrytis response regulation. These are now addressed in the revised manuscript and detailed in the four points (a-d) below.

a). A difference in the Botrytis strain or infection conditions used. The Mengiste et al., 2003 does not state which Botrytis strain was used. This is essential, as different Botrytis strains cause different symptoms. In our work, we have selected plant growth and infections conditions that result in a less aggressive infection, as discussed further below. Judging by the photos in the Mengiste et al., 2003 paper, a more aggressive infection system was used. Also, we now highlight in the Discussion of the revised manuscript several other differences in the methods used that may have also contributed to divergent results.

As the original Mengiste et al. paper relies on segregation analysis to conclude that bos1-1 was recessive, differences in the strength of infection caused by the Botrytis strain or infection conditions used could have led to mis-scored plants and distortion of the segregation ratios. Importantly, we are not able to evaluate these results critically, as no data are presented in support of this segregation analysis work, which is only described in the text in Mengiste et al. Also, the F2 population used for the segregation analysis was relatively small compared to many other studies.

b) The phenotype of the bos1-1/BOS1 F1 heterozygous plants, derived from crossing bos1-1 with wildtype. Mengiste et al, state that this F1 heterozygous plant "had a wild-type Botrytis-resistant phenotype," but also for this no data were shown. In our hands, the bos1-1/BOS1 F1 heterozygous plants displayed intermediate Botrytis susceptibility and wounding-induced spreading cell death (see Fig. 1 in the revised manuscript), consistent with our proposed function for bos1-1 as a gain-of-function allele.

c) Mengiste et al., also complemented the bos1-1 phenotypes with a genomic complementation construct containing BOS1 and its promoter. In this case, the results may have been limited by the technology and

expectations at the time. This paper states that 10 independent transformants were examined, but data presented from a single transgenic line to prove complementation. This practice is no longer acceptable today, due to the well known possibility for artifacts in some lines, which can give results that may not be representative. Importantly, the data provided was simply a photo that appears to be three leaves from a single infected plant, with no quantitative data or statistical analysis to support the conclusions.

The variability of transgenic lines is well illustrated in the new data that we added to our revised manuscript (see Fig. 5 in the revised manuscript). We expressed BOS1 under control of the 35S promoter and evaluated Botrytis lesion sizes, which correlated with the BOS1 transcript levels. However, within this analysis, there are several outliers, which illustrate the potential for spurious results in such systems and the need for evaluation of multiple transgenic lines for reliable results.

Finally, detailed inspection of the photos in Mengiste et al., figure 6, reveal that Botrytis infection of the complementation lines still display weak spreading cell death (clear chlorosis around the infection sites) not observed in the wildtype. This suggest that the bos1-1 lines transgenically expressing BOS1 under its own promoter were not fully complemented.

In our revised manuscript, we now also respectfully discuss the limitations of technology for a study published almost 20 years ago, compared to what is possible to do today (see lines 269-277; 350-352) .

d) We do not mention this in the manuscript; however, we would like to point out here that Mengiste et al., 2003 do not provide any evidence to explain how a T-DNA insertion in the 5'-UTR can simultaneously result in enhanced transcript accumulation and a loss of function in the gene. There is only the suggestion that "an aberrant transcript [is produced] that results either in no BOS1 being produced or in the production of a nonfunctional BOS1 protein." But no evidence was presented to support this suggestion. In our RNAseq data from bos1-1 (Fig. 4C), there is no evidence for alternative splicing or other changes to the BOS1 mRNA transcript, except for the increased transcript levels. Mengiste et al., also show that there is a 314-bp deletion in the BOS1 5' UTR between the T-DNA insertion site and the first exon (Figure 6a of Mengiste et al., 2003). Our resequencing data showed that there are two adjacent T-DNAs inserted in the 5'-UTR of BOS1. There is no evidence of the above mentioned deletion in the 5' UTR of BOS1 seen in these data (Fig. 4A). Additionally, we have assembled the BOS1 transcripts from our bos1-1 transcriptomic data and found the only genomic change detected in the BOS1 gene is the insertion of two T-DNAs (see Supplemental Fig. S1). The coding sequence of BOS1 was intact in bos1-1, however, compared to Col-0 reference, the 5' UTR is shorter as would be expected with transcription being initiated in the T-DNA insertion.

We identified a MAS promoter at the 3'-end of each T-DNA in bos1-1 (Fig. 4A), and they are known to drive cell death responsive gene expression. Accordingly, the MAS_{pro}:BOS1 lines we created fully reproduced the typical bos1-1 symptoms upon treatments of Botrytis and wounding (Fig. 4E). This supports that the MAS promoter in the T-DNA of bos1-1 is responsible for the bos1-1 symptoms rather than compromised function of BOS1. Our model, in which a gain of function semidominant trait is conferred by enhanced BOS1 expression driven by the MAS promoters in the T-DNAs, with BOS1 acting as a positive regulator of Botrytis-induced cell death, provides a clear explanation for the phenotypes of the bos1-1 allele, and all aspects of this are fully supported by clear and quantitatively measured evidence.

Point 4. Further complicating matters is the observation that your work doesn't measure Botrytis for dominance but instead measures the wounding cell death. That makes it somewhat difficult to assess your results, as the original paper shows recessive with one phenotype, Botrytis infection, whereas the letter shows semidominant with a different phenotype, wounding death, and claims the original paper was wrong. This is not sufficient evidence, as there are times when different phenotypes show different recessive/dominant nature. To justify a letter, the Botrytis assay would be necessary to measure for dominance to ensure you are comparing "apples to apples." There is also something potentially worrying about the Botrytis as it has not outgrown on Col-0, possibly suggesting the assays aren't working.

RESPONSE: We thank the editors for this excellent suggestion; we have performed the requested Botrytis assays (Figs, 1C, 3E and 4E). Similar to wounding analysis, the Botrytis sensitivity assays with all new genotypes in this manuscript, support the same conclusion that bos1-1 is a semidominant gain-of-function mutant.

Concerning the extent of Botrytis lesion outgrowth in Col-0 wildtype plants, we assure you that our Botrytis assays are working normally. Our lesions are smaller for several reasons. In the Mengiste et al., 2003 paper, more

aggressive infection conditions were used and the lesions were evaluated at 5 days post-infection (dpi). We have purposefully selected conditions that result in slow lesion growth, as these conditions make it easier to accurately measure the difference between wildtype Col-0 and bos1-1. In our experiments, lesion sizes were evaluated at 3 dpi. Lesions continue to grow days later than the 3 dpi that we used.

Point 5. Based on consultation with members of the editorial board and on reflection, we have determined that this work is not appropriate for resubmission as a “Letter to the Editor” and we will only consider the work as a regular research paper, as it is really extending the results of the Mengiste work with new data, and it needs to evaluated as primary research - not a short letter to the editor. For a primary research paper, considerably more data will be needed to make a more complete story.

The reference to “double mutants” is due to the observation that you have introduced CRISPR mutations in a genetic background already having the T-DNA. By comparison, the CRISPR mutation without the T-DNA doesn't seem to have an effect in your analysis. So there is something about having both mutations at once. It would be critical to understand why the CRISPR only shows a phenotype in the presence of the T-DNA. As mentioned in the decision letter, it is also critical to understand how changing the phenotype measurement may be identifying new aspects of the story, i.e. you appear to show the opposite of the original BOS1 paper by Mengiste et al., but you have changed the manner in which the phenotype is measured (the original paper shows recessive with one phenotype – the Botrytis assay – whereas your letter shows semidominant with a different phenotype, wounding death).

The original paper clearly established that the bos1 mutation behaves as a recessive phenotype with regards to Botrytis infection. Importantly, those authors further showed that this phenotype could be complemented by introduction of a wild-type genomic copy of the BOS1 gene. Those two pieces of data together are fully consistent with the original mutation being a loss-of-function. Your new data might suggest that it was not truly a loss-of-function that gave rise to the phenotype, which is interesting. However, you need to compare the same phenotype; i.e. with respect to Botrytis infection. Additionally, some explanation is required for how a "gain-of-function" phenotype can be complemented by introduction of a wild-type genomic copy; that is difficult to comprehend. So there is a considerable amount of additional data that would be needed for us to consider this as a full research paper that provides new/novel insight and the claims must be fully supported by the data. I hope this helps.

We would be willing to consider a resubmission that fully addressed these points by comparing phenotypes based on a Botrytis infection assay (not merely wounding) and offering discussion or an explanation for how a "gain-of-function" phenotype can be complemented by the introduction of a wild-type genomic copy. If these issues are addressed, it is still unclear whether this submission would be suitable as a letter to the editor (rather than a full primary research paper); we would need to conduct a full reassessment.

We look forward to your next submission and hope we will be able to render a more positive decision on future work.

TPC2021-RA-00979D Submission received Nov. 10, 2021

RESPONSE TO EDITORS: We submit the manuscript ‘BOS1 is a positive regulator of wounding induced cell death and plant susceptibility to Botrytis’ to be considered for publication at The Plant Cell as a regular research report. This is a resubmission of the manuscript TPC2021-LTE-00447, which was evaluated by members of the editorial board. The manuscript was not recommended for further review at that time, in light of the need for further development of the work.

We thank the editorial board for their insightful comments, which have led to significant improvements to our manuscript. In the intervening months, we have addressed the issues raised in the editorial review. This was accomplished in a thorough restructuring and rewriting of the manuscript, including the addition of a significant amount of new data. These changes are elaborated in the attached responses to the editorial board members’ comments.

TPC2021-RA-00979D 1st Editorial decision – *declined without external review* **Nov. 22, 2021**

Thank you for submitting your revised manuscript entitled "BOS1 is a positive regulator of wounding induced cell death and plant susceptibility to Botrytis" to The Plant Cell. Your submission has been evaluated by members of the editorial board, and we regret to inform you that we are not recommending that your manuscript in its current form proceed further in the review process. However, we would like to encourage you to resubmit a revised version of your manuscript to The Plant Cell, as we recognize the importance of your findings.

We have not made this decision lightly. Your submission was assessed at this stage by three editorial board members who judged that the work in its current form would still not be appropriate for The Plant Cell, and initiating additional review would only delay the eventual publication of your story.

To provide specific feedback: We had extensive discussion about this manuscript, recognizing the history of our discussions on it, from your earlier submission as a possible letter, and the changes that you made in response to our earlier comments. We agree that the newly-added information is useful but felt that the current evidence has still left some key gaps in understanding that need to be addressed. One is a direct test of whether the bos1-1 allele is recessive in your conditions. This is key, because if the bos1-1 shifts from recessive to dominant based on the environment, it would change the interpretation of the issue at hand. The conflict between the gain-of-function and recessive nature of the mutation needs some clarity. Additionally, if the interpretation is gain-of-function and given the data are that BOS1 is normally involved disease resistance, or, as the data in the manuscript suggest, maybe BOS1 is normally just involved in pollen formation -- and the gain-of-function is not indicative of normal function. These two questions are key to interpreting this new evidence.

If you can attend to these items we will be happy to reconsider the manuscript. We thank you for your interest in and support of The Plant Cell. We look forward to your next submission and hope we will be able to render a more positive decision on future work or the next version.

TPC2022-RA-00079D Submission received **Jan. 20, 2022**

RESPONSE TO EDITORS: We submit the manuscript 'BOS1 is a positive regulator of wounding induced cell death and plant susceptibility to Botrytis' to be considered for publication at The Plant Cell as a regular research report. This is a resubmission of the manuscripts TPC2021-LTE-00447 and TPC2021-RA-00979D, which were evaluated by members of the editorial board. The manuscript was not recommended for further review at that time, in light of the need for further development of the work.

We thank the editorial board for their insightful comments, which have led to significant improvements to our manuscript. In the intervening months, we have addressed the issues raised in the editorial review. The comments from TPC2021-LTE-00447 resulted in a thorough restructuring and rewriting of the manuscript, including the addition of a significant amount of new data. The comments from TPC2021-RA-00979D were addressed by investigation of the genetics in F2 segregation assays and multiple stress tests.

TPC2022-RA-00079D 1st Editorial decision – *revision requested* **March 11, 2022**

We have received reviews of your manuscript entitled "BOS1 is a positive regulator of wounding induced cell death and plant susceptibility to Botrytis." Thank you for submitting your best work to The Plant Cell. The editorial board agrees that the work you describe is substantive, falls within the scope of the journal, and may become acceptable for publication, pending revision and potential rereview.

We ask you to pay attention to the following points in preparing your revision:

Please consider and respond to all the points raised by the reviewers, which consists mostly of clarifications and interpretations. We should stress that we are reluctant to see manuscripts undergoing multiple rounds of revision and would be unlikely to offer you more than one chance to satisfy the reviewers.

RESPONSE TO EDITORS: We gratefully thank our editors and reviewers for their constructive comments and valuable suggestions. We have carefully addressed all the comments, which led to a significant improvement to our manuscript. Please see the details below.

In addition, we find that the writing suffers from some problems with grammar and awkward phrasing, and in general lacks clarity and conciseness. We strongly encourage the use of a professional editing service to ensure that the work is presented in the best manner and reaches the broadest possible audience. Several editing services have agreed to extend a discount to authors coming to their website via this ASPB page https://aspb.org/aspb-journal-submission-editing-services/.

RESPONSE TO EDITORS: The writing has been extensively edited and carefully proofread by all coauthors and several of our colleagues, including two native English speakers. These edits specifically addressed the issues mentioned above; grammar, phrasing, clarity, and conciseness. The manuscript now reads much better. Please note, these changes involve nearly the entire manuscript and are too numerous to elaborate here or indicate in the copy of the manuscript with the changes highlighted. We trust you understand.

TPC2022-RA-00079DR1 1st Revision received May 11, 2022

RESPONSE TO EDITORS: With this letter, we submit a revision of the manuscript TPC2022-RA-00079D entitled 'Ectopic expression of BOTRYTIS SUSCEPTIBLE1 in the bos1-1 allele reveals its function as a positive regulator of wound-induced cell death and plant susceptibility to Botrytis' to be further considered for publication at The Plant Cell.

We thank the editorial board and reviewers for their insightful comments, which have led to significant improvements to our manuscript. In the last months, we have addressed all questions raised by the editors and reviewers, including issues of formatting, language, checklist, scientific questions, and new experiments to better quantify wound-induced cell death. See the attached file "Response to reviewers" for the exact details. Further, as suggested by Reviewer 2, we looked into the history of the MAS promoter. The bos1-1 mutant is derived from the application of a T-DNA vector in which the MAS promoter drives expression of the BASTA resistance gene. The T-DNA mutant pool from which bos1-1 was isolated was also used to create the indexed SAIL T-DNA lines. Our manuscript now includes a discussion of potential caveats that should be considered when using T-DNA lines or other tools containing the MAS promoter, as the presence of this promoter might lead to unintended ectopic gene expression, similar to what we observed for bos1-1.

We contend that this study would command significant interest from researchers in the fields of development, cell death, and immune signaling. As such, it would be suitable for publication in The Plant Cell. We appreciate your consideration of our manuscript and look forward to hearing from you.

Reviewer comments on previous submission and **author responses**:

Reviewer #1 (Comments for the Author):

The authors do good work in characterizing the bos1-1 mutant plants as well as generating new lines of BOS1 by overexpression with the MAS promoter and by CRISPR mutation, and this helps to clarify the apparent contradiction previously observed between the phenotypes the bos1-1 line and the lines with TDNA insertions in the introns of BOS1. Overall, these new evidence helps to establish inheritance type of the bos1-1 allele originally described by Mengiste et al. and provides some insight to gene function, although not much mechanistic detail is added.

The work of Cui et al. shows that the original bos1-1 is inherited in a codominant fashion and that the original allele is a gain-of-function caused by overexpression in the TDNA line. Even though these two main findings are in contradiction the original work by Mengiste et al 2003, the evidence shown here supports the conclusions by the authors and they discuss what experimental procedures/conditions might be responsible for the contradictions between the two studies.

Major points

Point 1. Please clarify how the intragenic double mutant was created. In the Materials and methods section, the authors state bos1-1 is not amenable for Agrobacterium transformation, and so CRISPR mutations were introduced by Agrobacterium dipping in the WT background first and then crossed into the bos1-1 line. However, by this method it would be almost impossible to have the recombination necessary to create the new alleles described in Figure 4 of the manuscript. This would also help to understand the complementation line shown by Mengiste et al 2003.

RESPONSE: Indeed, bos1-1 is very sensitive to Agrobacterium, and in our hands all attempted transformations resulted in the bos1-1 plants dying after dipping. Thus Agrobacterium mediated transformation is not suitable for delivering Crispr vector directly to bos1-1. Instead, we used a strategy of crossing a Col-0 transformant bearing the Crispr/Cas9 vector as the pollen donor, with bos1-1 as the pollen acceptor. Importantly, this approach delivered a Crispr/Cas9 vector that was inserted at a chromosomal location not linked with the BOS1 locus. In the F1 plant, the Crispr/Cas9 vectors then functioned to modify the BOS1 gene and therefore did not rely on combining the modified BOS1 intron 2 with the T-DNA insertion of the bos1-1 allele. As the reviewer correctly pointed out, this would have required screening an impossibly large number of plants in order to identify such a rare intragenic crossover event. After segregation in the F2 population, bos1-1 homozygous plants were first selected by PCR screening, and then the Crispr/Cas9 knockouts were identified by Sanger sequencing. The reviewers comment has highlighted the problems with presentation of this process in our original submission. A better explanation of the methods used have been added to the Methods and materials section.

Point 2. As BOS1 is induced by JA treatment and the anther phenotype of the new BOS1 CRISPR alleles point to a role for BOS1 in JA signaling/metabolism. A brief discussion of this point should be included in the Discussion section.

RESPONSE: This is a good suggestion. We have added the following paragraph discussing the role of JA: "Another important regulator of anther development is jasmonic acid (JA). A characteristic phenotype of JA biosynthesis mutants and strong mutant alleles of the JA receptor COI1 (CORONATINE INSENSITIVE 1) is male sterility (Park et al., 2002; Mandaokar et al., 2003; Jewell and Browse, 2016). Two other MYB transcription factors, MYB21 and MYB24, act downstream from COI1 to regulate anther dehiscence (Song et al., 2011) and MYB24 acts redundantly with MYB108/BOS1 (Mandaokar and Browse, 2009). BOS1 expression is JA-dependent in flowers (Mandaokar and Browse, 2009); thus it is clear that multiple MYB transcription factors coordinate anther development. JA is also crucial for defense against insects and necrotrophic pathogen attack, and a characteristic phenotype for JA deficient or insensitive mutants is enhanced Botrytis lesion size (Thomma et al., 1998; Zhang et al., 2017). Botrytis infection leads to elevation of the BOS1 transcript level, which is fully dependent on JA signaling via COI1 (Mengiste et al., 2003). These similarities between JA-regulation of MYB transcription factors in both anthers and in Botrytis infection, suggest common components may be utilized for PCD regulation."

Reviewer #2 (Comments for the Author):

Importance of findings

Although much research was done in the past, regulation of cell death remains enigmatic in plants. This is exemplified by the remarkable spread of cell death lesions in the bos1-1 mutant. Cui et al. have made very important findings both previously and in this work to advance the field.

Quality of experiments

The experiments are of excellent quality with correct controls, statistics and interpretation. For example, I laud the honesty in highlighting data contrary to the hypothesis (35S overexpression lines #2 and #20 with high BOS1 expression, but no phenotype), but likely are the exceptions, as there is a clear correlation between expression and lesion diameter in the nine other overexpression lines.

Points in favor

The authors are getting to the bottom of the remarkable wound/pathogen-induced spread of cell death lesion in the bos1-1 mutant. For this they have generated new bos1 CRISPR alleles, performed some clever genetics, and had a key finding of the wound-inducible promoter in the original T-DNA of the bos1-1 mutant—key tools and experiments that were lacking previously. Through these experiments, the authors confirm the function of BOS1 in anther dehiscence. More importantly, the authors have done a fantastic job to show that the clear phenotypes of bos1-1 are due to an 'artefact' (this word doesn't justify the great value of this study). Namely, the wound-inducible overexpression of BOS1, which is caused by the introduction of the wound-inducible MAS1 promoter present in the T-DNA insertion (in the 5' UTR region) in the original bos1-1 mutant, is responsible for the cell death lesion spread.

Points detracting

It seems rather that deregulated expression of a PCD regulator from anther dehiscence has an accidental effect on pathogen/wound cell death. This is reflected in the lack of phenotypes during wounding/pathogen in the newly generated bos1 CRISPR alleles, absence of BOS1 induction after wounding in wild type (Fig. 4B) and the strongest expression of BOS1 during developmental phases (and to a lesser extent during (a)biotic stresses; Suppl. Fig 4). Still, the authors insist on connecting (a)biotic stress induced and developmental cell death (anther dehiscence). Therefore, the title of the manuscript is not justified ("BOS1 is a positive regulator of wounding induced cell death and plant susceptibility to Botrytis") and should instead be something in this trend: "Deregulated expression of BOS1 is an accidental positive regulator of wounding induced cell death and plant susceptibility to Botrytis in the bos1-1 allele."

Nevertheless, this deregulated expression could be a great tool in the future to learn more about the function of BOS1 in cell death spread, as it seems that different cell types (leaf vs anther) respond similarly to high levels of BOS1 expression, but leaf is easier to study (more tissue) than anther.

RESPONSE: These points are well received. We appreciate the reviewer's insights into the possible mechanisms of BOS1, and especially the idea that bos1-1 is a good tool to study cell death on the leaf. We agree and have integrated these suggestions into our manuscript.

Importantly, our work includes data that argues both for and against a role for BOS1 in stress responses in the leaves. We feel that a possible role of BOS1 in the leaf can not be fully excluded at this time. We contend that it is best to present both sides of this argument in the Discussion and highlight the need for further work to resolve this issue.

To do this, the following paragraph has been added: "There are two possible interpretations of the role of BOS1 in stress responses in leaves. On one side, ectopic overexpression of BOS1 driven by the MAS promoter may result in a leaf phenotype that is purely an artifact, suggesting that BOS1 is relevant only in the context of the flower, where it is naturally highly expressed. This argues against a role of BOS1 in stress-induced cell death in leaves and is supported by the lack of leaf cell death and stress phenotypes in bos1 loss-of-function mutants. On the other side, the ABA sensitivity phenotype of Crisper/Cas9-induced bos1 alleles suggests a role for BOS1 in regulating hormone responses in tissues outside the flower. Functions beyond the flower are also supported by the fact that BOS1 is stress inducible in leaves (Fig. S4). Additionally, leaves in the bos1-1 mutant and MAS_{pro}:BOS1 lines with elevated BOS1 transcript levels can undergo cell death (Figs 1, 4 and 5), which implies that the cell death signaling pathway(s) downstream of BOS1 are active in leaves. Considering these together, the possible relevance of BOS1 to Botrytis and other stress responses in the leaf cannot be excluded and further studies will be required to resolve this question."

We also agree with the need to revise the title, as suggested by Reviewer 2. Accordingly, the title has been changed to "Ectopic expression of BOTRYTIS SUSCEPTIBLE1 in the bos1-1 allele reveals its function as a positive regulator of wound-induced cell death and plant susceptibility to Botrytis.

Author actions necessary for acceptance:

Point 1. Fig. 2C - Why is the CRISPR line bos1-c3 not quantified?

RESPONSE: The data with bos1-c3 was quantified previously when the work with the other alleles was performed. We did not show the photos and data for bos1-c3 in the last version in an effort to reduce the size of the figure, as bos1-c3 exhibited the same behavior as bos1-c1 and -c2. Nonetheless, we have now added the bos1-c3 data back to the figure. Please see Figures 3B and C.

Point 2. What does the MAS promoter do in this T-DNA, and was this not known while generating the mutant (from literature)?

RESPONSE: Thanks for the suggestion to clarify the origin of the T-DNA in the bos1-1 mutant. We checked the original paper (McElver et al., 2001) that constructed the T-DNA insertion pool used by Mengiste et al 2003 in their screen leading to isolation of bos1-1. According to Materials and Methods in McElver et al., "Initial populations were generated with activation tagging vectors pPCVICEn4HPT (Hayashi et al. 1992) and pSKI015 (Weigel et al. 2000) containing multiple enhancers in the right border and elements for subsequent recovery of flanking sequences. Because this project did not emphasize the creation of gain-of-function mutations, most insertion lines were made with simple T-DNA vectors (pCSA104, pDAP101, pCSA110) with a BASTA-resistance gene, plasmid rescue element, and minimal borders designed for production of gene knockouts."

We checked the original papers for the enhancer-containing vectors of pPCVICEn4HPT (Hayashi et al., 1992) and pSKI015 (Weigel et al., 2000), and found that both of them contain tandem CaMV 35S enhancer sequences, but not the MAS promoter.

To see if the other vectors (pCSA104, pDAP101, pCSA110) used for construction of the T-DNA insertion pool contain the MAS promoter, we obtained the sequence information via http://seedgenes.org/FlankingSequence.html. All three vectors contain the MAS promoter sequence (Supplemental Figure 8).

The original purpose of the MAS promoter in these vectors was drive expression of the BlpR gene, which confers Basta resistance. We suspect that McElver et al., 2001 might not have been aware of the potential for the MAS promoter to activate genes outside of the vectors (pCSA104, pDAP101, pCSA110), as they claimed in their paper that these vectors contain no activation promoters and their initial purpose was to use these vectors to create knockout mutants.

Notably, the T-DNA insertion pool made by McElver et al., 2001 was used to create the widely used SAIL T-DNA lines, which were generously made available to the community through the ABRC and NASC Arabidopsis Stock Centers. See Sessions et al., 2002, for a description of the SAIL line T-DNA mutant collection, including the use of pCSA110 and pDAP101.

Our finding that the SAIL lines contain the MAS promoter has broad implications for the use of SAIL T-DNA lines in reverse genetic studies. Similar to the situation with bos1-1, there might be other situations with this MAS promoter containing T-DNA inserted in the promoter region that could cause overexpression rather than knockouts. We added a new section at the end of our manuscript, in which we advise caution in the use of SAIL and other T-DNA lines containing the MAS promoter.

We are grateful that the reviewer raised this question, which led to the discovery of the important caveat that some T-DNA lines contain the enhancing MAS promoter. We consider this as an additional valuable contribution from our manuscript to the plant research community.

McElver, J. et al. (2001). Insertional mutagenesis of genes required for seed development in Arabidopsis thaliana. Genetics 159: 1751–1763.

Hayashi, H., Czaja, I., Lubenow, H., Schell, J., and Walden, R. (1992). Activation of a Plant Gene by T-DNA Tagging: Auxin-Independent Growth in Vitro. Science 258: 1350–1353.

Sessions, A. et al. (2002). A High-Throughput Arabidopsis Reverse Genetics System. Plant Cell 14: 2985–2994.

Weigel, D. et al. (2000). Activation Tagging in Arabidopsis. Plant Physiology 122: 1003–1014.

Point 3. Change of the title and discussion to reflect the 'accidental nature' of BOS overexpression in the bos1-1 mutant.

RESPONSE: As stated above, we have changed the title in line with the reviewer's suggestion and hypothesis of the accidental nature of BOS1 overexpression in the bos1-1 mutant has been integrated into the discussion. Please see responses 1 and 2 above for more details.

Reviewer #3:

My concerns have been sufficiently addressed, thank you.

I agree that the extra paragraph on the potential problems with the MAS promoter in existing T-DNA collections is warranted.

TPC2022-RA-00079DR1 2nd Editorial decision – *acceptance pending* **May 31, 2022**

We are pleased to inform you that your paper entitled "Ectopic expression of BOTRYTIS SUSCEPTIBLE1 in the *bos1-1* allele reveals its function as a positive regulator of wound-induced cell death and plant susceptibility to Botrytis" has been accepted for publication in *The Plant Cell*, pending a final minor editorial review by journal staff. At this stage, your manuscript will be evaluated by a Science Editor with respect to scientific content presentation, compliance with journal policies, and presentation for a broad readership.

Final acceptance from Science Editor **July 13, 2022**
